# Structural integrity of the PCI domain of eIF3a/TIF32 is required for mRNA recruitment to the 43S pre-initiation complexes

**DOI:** 10.1093/nar/gkt1369

**Published:** 2014-01-13

**Authors:** Sohail Khoshnevis, Stanislava Gunišová, Vladislava Vlčková, Tomáš Kouba, Piotr Neumann, Petra Beznosková, Ralf Ficner, Leoš Shivaya Valášek

**Affiliations:** ^1^Department of Molecular Structural Biology, Institute for Microbiology and Genetics, George-August University, Goettingen, Germany, 37077 and ^2^Laboratory of Regulation of Gene Expression, Institute of Microbiology ASCR, Videnska 1083, 142 20 Prague, the Czech Republic

## Abstract

Transfer of genetic information from genes into proteins is mediated by messenger RNA (mRNA) that must be first recruited to ribosomal pre-initiation complexes (PICs) by a mechanism that is still poorly understood. Recent studies showed that besides eIF4F and poly(A)-binding protein, eIF3 also plays a critical role in this process, yet the molecular mechanism of its action is unknown. We showed previously that the PCI domain of the eIF3c/NIP1 subunit of yeast eIF3 is involved in RNA binding. To assess the role of the second PCI domain of eIF3 present in eIF3a/TIF32, we performed its mutational analysis and identified a 10-Ala-substitution (Box37) that severely reduces amounts of model mRNA in the 43–48S PICs *in vivo* as the major, if not the only, detectable defect. Crystal structure analysis of the a/TIF32-PCI domain at 2.65-Å resolution showed that it is required for integrity of the eIF3 core and, similarly to the c/NIP1-PCI, is capable of RNA binding. The putative RNA-binding surface defined by positively charged areas contains two Box37 residues, R363 and K364. Their substitutions with alanines severely impair the mRNA recruitment step *in vivo* suggesting that a/TIF32-PCI represents one of the key domains ensuring stable and efficient mRNA delivery to the PICs.

## INTRODUCTION

Protein biosynthesis begins with formation of the 43S pre-initiation complex (PIC) consisting of the small ribosomal subunit, Met-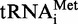 [in the form of the ternary complex (TC) together with the eukaryotic initiation factor eIF2 in its GTP form], eIF1A, eIF1, eIF3 and eIF5 [reviewed in (1)]. In the following step, messenger RNA (mRNA) is loaded onto the 43S PIC with help of eIF3, poly(A)-binding protein (PABP) and the eIF4F factors bound to its 5′ 7-methylguanosine cap structure, producing the 48S PIC. Subsequently, usually the first AUG codon in the mRNA’s 5′ UTR is recognized as the start site during the successive movement—called scanning—of the 48S PICs downstream from the cap. On AUG recognition, eIF2 in its GDP form is released from the ribosome along with several other eIFs, the 60S subunit joins the 40S•Met-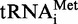
•mRNA complex in a reaction promoted by eIF5B and the resulting 80S initiation complex is on ejection of the remaining eIFs—with the exception of eIF3 and perhaps also eIF4F (2,3)—prepared for elongation.

In prokaryotes, the mRNA recruitment step is well defined and involves a direct RNA–RNA interaction between the 3′-end of 16S rRNA of the small ribosomal subunit and a specific Shine–Dalgarno sequence located in the 5′-end of mRNAs to allow direct positioning of the AUG start codon into the ribosomal P-site. In contrast, eukaryotic mRNAs do not contain anything like Shine–Dalgarno, initiating AUG is usually tens or even hundreds of nucleotides downstream from the cap and their recruitment to the 43 PICs requires the concerted action of several eIFs. This step, along with the subsequent scanning (base-to-base inspection of the mRNA’s 5′ UTR), represents one of the least understood reactions in the entire eukaryotic initiation pathway. In the current textbook view, eIF3, PABP and the eIF4F complex (comprising the molecular scaffold eIF4G, to which the cap-binding protein eIF4E and the DEAD-box RNA helicase eIF4A bind) are proposed to be responsible for mRNA loading to the 43S PICs [reviewed in (1)]. Besides eIF4E and eIF4A, the scaffold eIF4G also interacts with PABP and, in mammals, with eIF3 (4–8). The eIF4G–eIF3 interaction has been long believed to serve as the umbilical cord connecting the eIF4F•mRNA and 43S complexes, thus mediating formation of the 48S PIC, at least in mammals [in budding yeast, where the eIF3-binding domain in eIF4G is not evident, the direct eIF3–eIF4G interaction has never been detected (9)]. However, recent *in vivo* findings suggested that not only in yeast but probably also in mammals the mRNA recruitment step might be a lot less dependent on the direct eIF4G–eIF3 contact than it has been believed so far (10–13). Consistently, recent *in vivo* and *in vitro* studies in yeast indicated that eIF3 plays a more critical role in mRNA recruitment than eIF4G (14,15). Certainly, a more systematic approach is needed to fully understand this critical initiation step that is also known to serve as one of the two major targets for the general translational control [reviewed in (16)]. The first attempt in this direction has been recently made by identifying several mutations occurring in the j/HCR1-like domain (HLD) of the eIF3a/TIF32 subunit that, besides other effects on general initiation steps, also partially affected efficiency of the model mRNA recruitment to 40S ribosomes (17).

**Figure 1.**
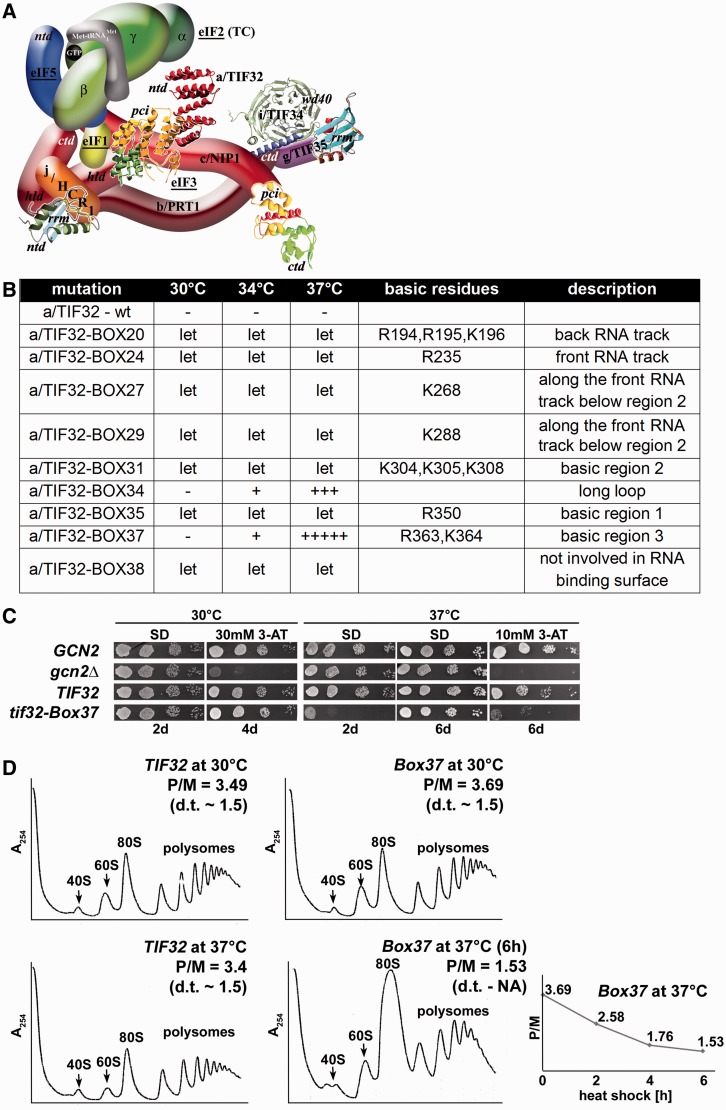
The 10-Ala *Box37* substitution of α-helix 4 of the HB subdomain in the a/TIF32-PCI domain reduces the translation initiation rates at the restrictive temperature. (**A**) A 3D model of eIF3 and its associated eIFs in the MFC [adapted from (25)]. *ntd*, N-terminal domain; *ctd*, C-terminal domain; *hld*, HCR1-like domain; *rrm*, RNA recognition motif; *pci*, PCI domain; TC, ternary complex. The nuclear magnetic resonance structure of the interaction between the RRM of human eIF3b (green and light blue) and the N-terminal peptide of human eIF3j (yellow) (24), the nuclear magnetic resonance structure of the C-terminal RRM of human eIF3g (19), the X-ray structure of the yeast i/TIF34–b/PRT1-CTD complex (green versus blue) (25), the X-ray structure of the a/TIF32-PCI (yellow and green) and structural prediction of the N-terminal domain of a/TIF32 (red) (both this study) and the 3D homology model of the c/NIP1-CTD (34) were used to replace the original schematic representations of the corresponding molecules. (**B**) Summary of the phenotypic analysis of Ala substitutions in consecutive blocks of 10 residues between amino acids 191 and 400 (dubbed Boxes 20–40). The indicated mutant alleles were introduced into the H477 strain containing a/TIF32 under control of *MET3* promoter and tested for growth in the presence of methionine. Alleles showing the Ts^−^ or Slg^−^ or lethal (Let) phenotypes are listed; the ± scoring system defines the wt as ‘–’ and no growth as 5+. (**C**) The *tif32-Box37* mutation imparts the Ts^−^ and Gcn^−^ phenotypes. YBS52 (*GCN2 a/tif32Δ*) was transformed with YCplac111-based plasmids carrying either *a/TIF32* wt or *a/tif32-Box37* mutant alleles, and the resident YCpTIF32-His-U plasmid was evicted on 5-FOA. The resulting strains, together with isogenic strains H2880 (*GCN2 a/TIF32*; row 1) and H2881 (*gcn2Δ a/TIF32*; row 2) transformed with empty YCplac111 vector, were then spotted in four serial dilutions on SD or SD containing 10- or 30-mM 3-AT and incubated at 30°C or 37°C for up to 6 days. (**D**) The *tif32-Box37* mutation reduces the translation initiation rates at the restrictive temperature. Polysome profiles of *TIF32* wt and *tif32-Box37* mutant strains described in section B were recorded from cells cultured in YPD medium at 30°C or heat shocked at 37°C for indicated time points and subsequently treated with cycloheximide before harvesting. Whole-cell extracts were separated by velocity sedimentation through a 5–45% sucrose gradient centrifugation at 39 000 rpm for 2.5 h. The gradients were collected and scanned at 254 nm to visualize the ribosomal species. Positions of 40S, 60S and 80S species are indicated by arrows, and polysome to monosome (P/M) ratios and doubling times (d.t.) are given above the profiles. The plot indicates P/M ratios collected at various time points of heat shock.

Despite the critical importance of the multisubunit eIF3 complex in translation—in addition to the mRNA recruitment, it also promotes the TC recruitment, scanning, AUG recognition and strikingly also translation termination (17–27)—a high-resolution structural picture of both yeast and mammalian eIF3 as whole remains elusive. Only partial either crystal or nuclear magnetic resonance structures were solved for a handful of domains of some of the eIF3 subunits, alongside the low-resolution Cryo-EM structure of the 13-subunit human eIF3 (28,29). In particular, the structures of RNA recognition motif (RRM) of yeast eIF3b/PRT1 subunit (30) as well as its human ortholog alone (31) or bound to the N-terminal peptide of eIF3j/HCR1 (24), the seven-bladed β-propeller of the WD40 eIF3i/TIF34 subunit bound to the extreme C-terminal α-helix of b/PRT1 (25), the RRM of human eIF3g/TIF35 (19) and finally the atypical PCI domain-containing human eIF3k subunit (32) have been determined at atomic resolution so far. The PCI domain is a conserved bipartite domain comprising the helical bundle (HB) and winged-helix (WH) subdomains initially identified as the principal scaffold for the multisubunit 26S proteasome lid, signalosome (CSN) and eIF3 complexes (33). Yeast eIF3 contains two PCI domains, one at the N-terminus of its eIF3a/TIF32 subunit and the other at the C-terminus of eIF3c/NIP1. In fact, the 3D architecture of the eIF3c/NIP1-PCI was recently predicted *in silico* and proposed to fold into a typical bipartite arrangement (34). Most of these partial structures and the latter prediction were incorporated into a structural model of yeast eIF3 shown in Figure 1A. Importantly, the latter report also revealed for the first time that the c/NIP1-PCI domain, this typical protein–protein interacting mediator, is capable of strong binding to RNA. Later, yeast SAC3 and THP1 components of the TREX-2 transcription–export complex, both containing the PCI domain, were shown to have their WH subdomains juxtaposed in a way forming a platform that is also capable of nucleic acid binding (35).

To analyze the function of the N-terminus of a/TIF32 harboring the second PCI domain of yeast eIF3, we undertook a systematic mutagenesis of a large N-terminal segment of a/TIF32 including an N-terminal part of the PCI domain. We show that a 10-alanine substitution of residues 361–370 (designated Box37) markedly affects growth and translation initiation rates at the elevated temperature. Structural investigation of the a/TIF32-PCI domain showed that it consists of two intimately connected subdomains: an N-terminal right-handed helical domain capped by a C-terminal winged helix domain. Furthermore, we provide evidence that specifically the PCI domain of a/TIF32 (i) mediates its binding to the eIF3c/NIP1 subunit of eIF3 and (ii) that the entire N-terminal half of a/TIF32 interacts with RNA, as proposed before (2,37). Interestingly, neither the N-terminal domain nor the PCI domain alone is sufficient for this binding; however, they are both required. In addition to that, we show that α-helix 4 of the HB subdomain, and in particular its two consecutive basic residues R363 and K364, strongly contributes to mRNA recruitment of the model mRNAs to the 43S PICs *in vivo*. Although R363 is also required for stabilization of the HB fold, K364 is fully exposed to the solvent with no structural role. Further analysis of the crystal structure and the *in silico*-generated structure of the helical region preceding this domain revealed the dominance of continuous positively charged areas over the surface of the a/TIF32 N-terminal half, proposing a molecular basis for its role in RNA recruitment. In support, 10-Ala substitutions of these basic patches resulted in lethality. Taken together, because our mutants in Box37 impair the mRNA loading process as the only detectable defect *in vivo*, we propose that one of the key roles of this well-defined domain is, besides building the eIF3 core, to contact mRNAs in a non-specific manner and promote their delivery to and/or stable binding on the small ribosomal subunit. Hence, the a/TIF32-PCI domain is yet another PCI domain demonstrated to be involved in RNA binding.

## MATERIALS AND METHODS

### Yeast strains, plasmids and biochemical methods

Lists of strains, plasmids and oligonucleotides used in this study (Supplementary Tables S1–S3), details of their construction, as well as description of all well-established biochemical assays used throughout the study can be found in the Supplementary Data that are available at NAR online.

### Cloning, expression, purification and crystallization of the a/TIF32^276^^−494^ domain and crystal structure determination

a/TIF32^276^^−494^ was cloned into a pGEX6P1 vector, expressed in *Escherichia coli* Rosetta2 (DE3) cells and purified via affinity and size-exclusion chromatography (SEC) (detailed purification schemes of proteins used in this study are provided in Supplementary Methods). Crystals were grown at 4°C in sitting drop vapor diffusion plates by mixing 0.8 μl of crystallization solution [100 mM HEPES, pH 7, 12% (w/v) PEG 2000, 80 mM MnCl_2_] and 1.2 μl of protein solution (at a concentration of 6 mg/ml in the gel filtration buffer). Crystals were cryoprotected by soaking in reservoir solution supplemented with increasing concentration of PEG400 to 40% (w/v) and flash frozen in liquid nitrogen before data collection.

X-ray diffraction images were collected at 100 K at beamline 14.1 [BESSY, Berlin, Germany; (37)] equipped with a MAR Mosaic 225 mm CCD detector (Norderstedt, Germany). The oscillation images were indexed, integrated and merged using the XDS package (38,39) to the final resolution of 2.65 Å for the native and to 2.79, 2.81 and 2.97 Å for Se-Met derivative crystal (peak, inflection and remote data sets, respectively). The crystal structure of a/TIF32 was solved by means of single wavelength anomalous dispersion SAD using the Se-Met data set at the peak wavelength in SHARP/autoSHARP (40). Within autoSHARP, the heavy atom search was performed by SHELXD (41) and resulted in localizing four heavy atom positions that were further refined using SHARP followed by density modification in Solomon (42) and automatic model building in Arp/wARP (43). Structure determination and refinement procedure are discussed in detail in Supplementary Methods.

### RNA-binding assay

RNA-binding studies were performed in 1× recon buffer (44). Two micromolars of *DAD4* RNA (see Supplementary Methods for transcription procedure) was mixed with 10 µM a/TIF32^FL^, a/TIF32^1^^−494^, a/TIF32^276^^−494^, GST-a/TIF32^1^^−276^ and GST in a total volume of 10 µl. After a 30-min incubation on ice, samples were mixed with native loading dye and applied to a 0.8% agarose gel supplemented with GelRed (Biotium). Electrophoresis was performed at 90 V for 30 min on ice using pre-cooled 1 × Tris acetate + ethylenediaminetetraacetic acid running buffer. The gel was subsequently exposed to ultraviolet-light to visualize nucleic acids.

### Binding of the a/TIF32^276^^−494^ domain to c/NIP1 by analytical SEC

Interaction studies were performed on an analytical Superdex 200 (10/300) column (GE Healthcare) in a buffer containing 150 mM KCl, 10 mM Hepes, pH 7.5, and 5% glycerol. Fifty micrograms of c/NIP1 (0.5 nmol), b/PRT1 (0.5 nmol) or a/TIF32^276^^−494^ (2 nmol) in the total volume of 500 µl was loaded on the column separately or after being incubated together for 30 min.

### Isothermal titration calorimetry

c/NIP1 and a/TIF32^276–494^ were extensively dialyzed against ITC buffer (150 mM KCl, 10 mM Hepes, pH 7.5, 5% glycerol) and concentrated to 10 and 100 µM, respectively. The experiment was performed on a VP-ITC calorimeter (Microcal, USA). Twenty microliter aliquots of a/TIF32^276–494^ were injected into the cell containing c/NIP1 every 40 s, during which the titration peak returned to the base line. Separately, seven injections of the same concentration of a/TIF32^276–494^ into the buffers were performed under the same conditions to determine the heat of dilution. The titration data were analyzed using the ORIGIN software to calculate the thermodynamics parameters.

## RESULTS

### A specific 10-alanine substitution (*Box37*) in the a/TIF32-PCI domain results in temperature sensitivity and reduces translation initiation rates

Domain prediction programs detect two PCI domains in the yeast eIF3 complex. The first one, located at the C-terminus of c/NIP1, was previously implicated in RNA binding as the first PCI domain known to do so (34). Here we sought to investigate the role of the second PCI domain of eIF3 occurring in the N-terminal half of a/TIF32. We have recently demonstrated that the proteolytic digestion of a/TIF32 generates a fragment harboring its first 494 residues (45) suggesting that the entire a/TIF32 N-terminal half, including the PCI domain, is folded into a higher order structure. Earlier we also showed that the a/TIF32 region corresponding to amino acid (aa) residues 201 through 400, encompassing a part of the a/TIF32-PCI domain, interacts with the small ribosomal protein RPS0A (46) situated on the solvent-exposed side near the 40S mRNA exit channel (47).

To show whether the a/TIF32-PCI domain is involved in RPS0A binding and to identify specific residues that mediate this contact, we introduced Ala substitutions in consecutive blocks of 10 residues between amino acids 191 and 400 (dubbed Boxes 20–40) and tested them for growth phenotypes, first in a yeast strain containing a/TIF32 under control of *MET3* promoter. Several of these Ala substitutions led to lethal phenotypes (Figure 1B; see also below) in accord with our earlier observation that deletion of the first 350 residues of a/TIF32 is deleterious (18). One of these substitutions, Box37 (residues 361–370), produced strong temperature-sensitive (Ts^−^) and Gcn^−^ phenotypes (Figure 1C), which we could analyze further for its primary defect—the Gcn^−^ (general control non-derepressible) phenotype indicates an impairment of the *GCN4* induction (see below). (Box34 [residues 331–340] also showed the Ts^−^ phenotype; however, as it was rather mild, we did not pursue this mutant any further). Careful inspection of polysome profiles carried out at the non-permissive temperature at three time points following the temperature up-shift clearly suggests that the *Box37* mutation specifically affects translation initiation rates as the run-off 80S couples accumulate at the expense of heavy polysomes, which is displayed by ∼2.2-fold decrease in the polysome to monosome (P/M) ratio (Figure 1D).

**Figure 2.**
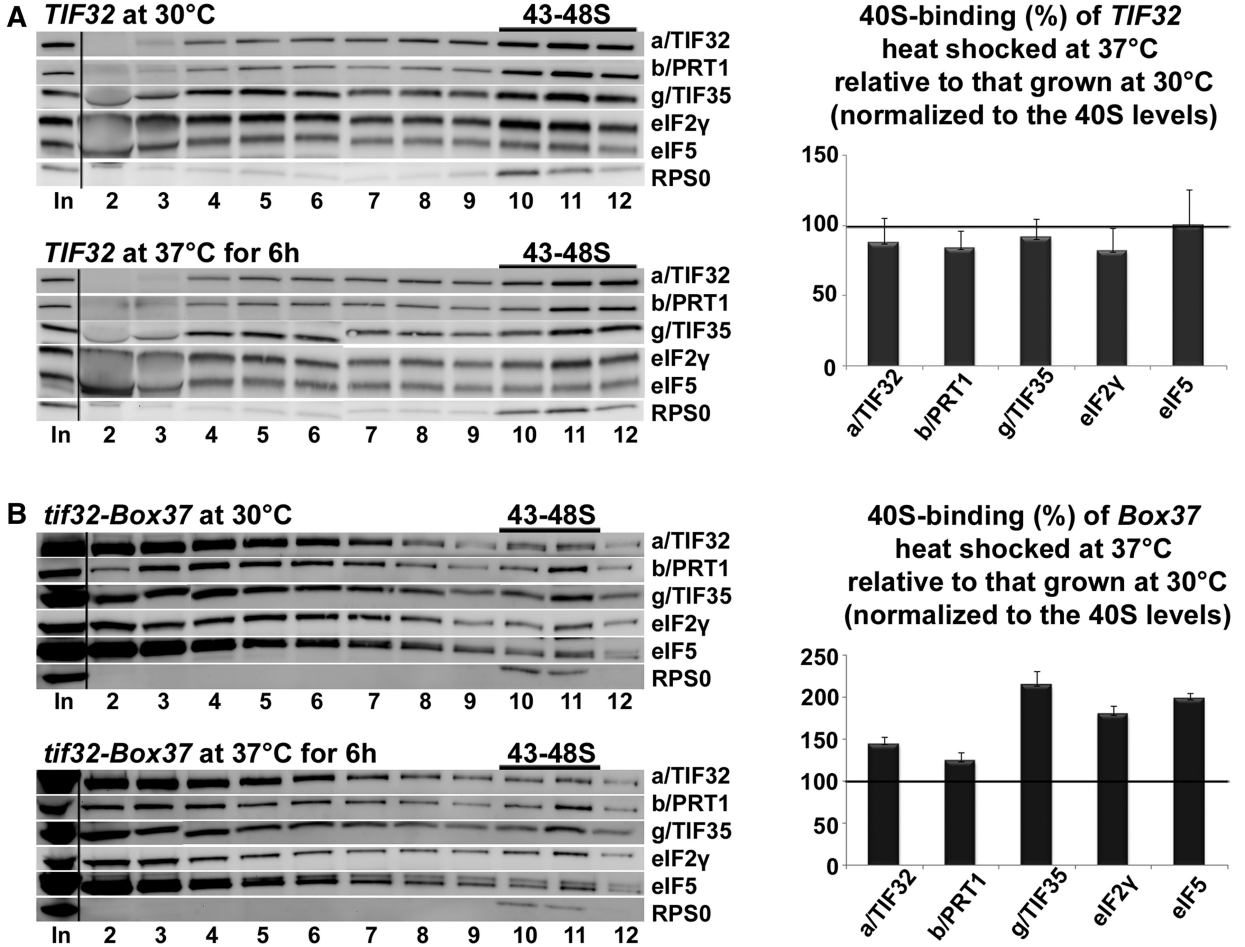
The *tif32-Box37* substitution results in accumulation of the 43S PICs. The *tif32-Box37* mutant strain (**B**) and its corresponding wt (**A**) described in Figure 1C were grown in YPD medium at 30°C or heat shocked at 37°C for 6 h and cross-linked with 2% HCHO before harvesting. Whole-cell extracts were prepared, separated on a 7.5–30% sucrose gradient by centrifugation at 41 000 rpm for 5 h and 5% of each fraction was loaded on the gel and subjected to western blot analysis. ‘In’ shows 5% input. Proportions of the 40S-bound proteins relative to the amount of 40S subunits were calculated using Quantity One software (BioRad) from at least three independent experiments. The resulting values obtained with cells grown at 30°C were set to 100% and those obtained with heat-shocked cells were expressed as percentages of the 30°C-grown cells in the histogram on the right (SDs are given).

Next we examined the potential effect of *Box37* on the direct interaction of the 201–400 subdomain of a/TIF32 with RPS0A in an *in vitro* binding assay as well as on the integrity of native eIF3 with eIFs 1, 2 and 5 into the ribosome-free multifactor complex (MFC) *in vivo*. Neither was found dramatically affected by this mutation (data not shown) suggesting that *Box37* did not markedly affect the mutant protein fold, if at all (see also ‘Discussion’ section), and also that it is not the critical anchor point between the a/TIF32-NTD and RPS0A. Despite that we still asked whether this mutation may interfere—perhaps in some indirect way—with the initial assembly of eIFs on the 40S ribosomes. Toward this end, we analyzed the distribution of selected initiation factors in *Box37* cells grown at 30°C and subsequently subjected to heat shock at 37°C for 6 h using formaldehyde cross-linking of living cells followed by high velocity sedimentation of whole-cell extracts (WCE) in sucrose density gradients (48). No reduction in the 40S-associated amounts of selected eIF3 subunits as well as eIF2 and eIF5 was observed in heat-shocked cells (Figure 2B). On the contrary, when normalizing to the levels of 40S species, we detected ∼1.5-fold accumulation of the latter eIFs in heat-shocked cells compared with cells grown at the permissive temperature. Wild-type (wt) cells showed rather decreased, but definitely not increased, amounts of eIFs in 40S fractions on heat shock (Figure 2A). This suggests that the rate-limiting step that is impaired in *Box37* follows the 43S PIC assembly.

### The *tif32-Box37* mutation imparts severe Gcn^−^ phenotype that is not caused by post-assembly defects in the initiation pathway

To examine what step is affected by *Box37*, we used the translational control mechanism of *GCN4*, which depends on four short upstream open reading frame (uORFs) found in its mRNA leader and has been adapted to serve as an experimental tool for monitoring various translational steps [reviewed in (49)]. The expression of *GCN4*, a transcriptional activator of many biosynthetic genes, is delicately regulated in a nutrient-dependent manner by the GCN2 kinase. Under nutrient-replete conditions, the kinase is inactive and the *GCN4* expression is repressed. On amino acid starvation, GCN2 becomes activated and derepresses *GCN4* synthesis by reducing the steady-state levels of the TC. Mutants defective in the TC formation and/or its recruitment to the 40S subunit mimic starvation conditions and constitutively derepress *GCN4* even under nutrient-replete conditions, producing the Gcd^−^ phenotype. Conversely, mutants that fail to derepress *GCN4* under starvation conditions provoke the Gcn^−^ phenotype, which typically signals defects in the steps following assembly of 43–48S PICs, such as processivity of scanning, AUG recognition or subunit joining.

The fact that *Box37* displayed the severe Gcn^-^ phenotype at the restrictive temperature (Figure 1C), which is characterized by a failure to grow in the presence of an inhibitor of histidine biosynthetic genes—3-aminotriazole (3-AT), prompted us to use a battery of *GCN4-lacZ* reporter constructs with specific modifications in the *GCN4* mRNA leader. These have been successfully used in the past to reveal malfunctioning in scanning processivity, scanning rates, stringency of AUG selection or in subunit joining (2,17,19,20,24,25,50). First, we tested the wt *GCN4-lacZ* reporter plasmid to verify the true nature of the Gcn^−^ phenotype in *Box37* cells and found that the degree of *GCN4-lacZ* induction was ∼4-fold reduced in mutant (showing rather low ∼2-fold induction) versus wt (showing standard ∼8-fold induction) cells. This clearly indicates that the inability to compete with 3-AT on plates is a direct consequence of an insufficient *GCN4* derepression in response to 3-AT. Surprisingly, detailed analysis of all potential post-assembly defects described above did not reveal any clearly distinguishable defects; we only noticed that most of the constructs showed generally reduced β-galactosidase activities in mutant *versus* wt strains. Taking this into consideration, plus the fact that yeast eIF3 has been recently implicated in serving as one of the major factors ensuring stable mRNA binding to 40S ribosomes (14,15,17), low levels of wt *GCN4-lacZ* induction could be explained by a failure to recruit or stably accommodate mRNAs on the 43S PICs to form the 48S PICs poised for scanning. Interestingly, a similar phenotype was only recently reported for the eIF4G mutant *tif4632-7R* (51). In fact, this particular defect could also explain increased amounts of eIFs on 40S ribosomes that we observed in heat-shocked mutant cells (Figure 2B), as an increased number of 40S species bound only by the initial set of factors promoting the Met-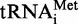 recruitment would be expected to accumulate in such defective cells. In further support, the ‘40S’ peak in polysome profiles recorded with heat-shocked *Box37* cells is divided into two equally large sub-peaks, whereas only one dominant peak (the heavier one) occurs in wt cells (Figure 1D). In our previous work (9), we proposed that the lighter sub-peak corresponds to ‘naked’ 40S ribosomes combined with the 43S PICs and the heavier one to the 48S PICs. Hence, occurrence of two 40S sub-peaks in the heat-shocked mutant cells could be explained by slower conversion of 43S PICs into 48S PICs due to impaired mRNA recruitment (see below). To shed light on the molecular mechanism by which a/TIF32-PCI domain would facilitate the recruitment of mRNA to the ribosome, we set forth to determine its 3D structure at atomic resolution.

### Purification, crystallization and structure determination of the a/TIF32 PCI domain

The N-terminal region of a/TIF32 was previously identified as a stable protein domain (45). As mentioned previously, this region is predicted to harbor a PCI domain preceded by several α-helices. PCI domains are made up of two subdomains: N-terminal helical repeats followed by a C-terminal-winged helix motif. While the C-terminal winged helix motif can be fairly accurately predicted, the prediction of the exact length of the N-terminal subdomain of a/TIF32 turned out to be difficult. Because we did not obtain crystals of the entire N-terminal domain of a/TIF32, we systematically removed helices from the N-terminus, keeping the C-terminus at residue 494. However, most of the constructs led to insoluble proteins. The most soluble fragment was obtained by removing the first 275 residues (Supplementary Figure S1). This fragment was crystallized in a solution containing 100 mM HEPES, pH 7, 12% PEG 2000 (w/v), 80 mM MnCl_2_. Crystals grew at 4°C to the maximum size in 1 week. Cryoprotection was achieved by soaking crystals in several drops containing the crystallization solution and increasing concentration of PEG400. Sudden increase of the PEG400 concentration caused cracks in the crystal and had a severe effect on the diffraction quality. The crystal structure of a/TIF32^276-494^ was solved by single wavelength anomalous dispersion using a selenomethionine (SeMet) derivative and refined to the final resolution of 2.65 Å. The asymmetric unit (AU) is composed of two molecules of a/TIF32^276^^−494^, which corresponds to a solvent content of 45%. Although the first molecule in the AU (chain A in the pdb file) is almost complete, the second molecule is only partially defined in the electron density map. The crystallographic statistics for data processing and refinement are presented in Table 1.

**Table 1.** Data collection, phasing and refinement statistics

	Native	Peak	Inflection	High remote	Low remote
**Data collection**					
Wavelength (Å)	0.933400	0.979540	0.979690	0.977260	0.982450
Resolution (Å)	50.00–2.65 (2.75–2.65)	50.00–2.79 (2.89–2.79)	50.00–2.81 (2.91–2.81)	50.00–2.97 (3.07–2.97)	50.00–2.97 (3.07–2.97)
Space group	I 2_1_ 3	I 2_1_ 3	I 2_1_ 3	I 2_1_ 3	I 2_1_ 3
Cell parameters:					
a = b = c (Å)	137.137	137.63	137.99	138.06	137.75
α = β = γ = 90 (°)					
R_merge_ (%)	5.9 (78.0)	10.4 (83.4)	10.8 (88.7)	8.8 (60.2)	10.2 (71.7)
*I*/δ*I*	17.76 (1.83)	21.44 (2.16)	21.7 (2.0)	23.21 (2.77)	21.36 (2.53)
*CC_(1/2)_* (%)	99.9 (66.2)				
Completeness (%)	99.5 (99.8)	99.6 (96.5)	99.7 (97.3)	99.9 (100)	99.8 (99.9)
Redundancy	3.67 (3.71)	4.41 (3.48)	4.44 (3.58)	4.93 (4.92)	3.89 (3.88)
Anomalous correlation		56 (11)	35 (7)	35 (4)	16 (11)
Mean anomalous difference (SigAno)		1.724 (0.849)	1.285 (0.794)	1.269 (0.806)	0.997 (0.845)
**Refinement**					
Resolution (Å)	48.48–2.65 (2.75–2.65)				
Number of reflections	12582				
*R*_work_/*R*_free_ (%)	24.89/28.61				
Number of atoms					
Protein	3037				
*B*-factors (Å^2^					
Protein	118.70				
Entity nr. 1/2	81.5/168.8				
R.m.s deviations					
Bond lengths (Å)	0.006				
Bond angles (°)	1.26				
PDB code	4K51				

The CC_1/2_ is the correlation coefficient between two randomly selected half data sets as described in (52).

### Crystal structure analysis

**Figure 3.**
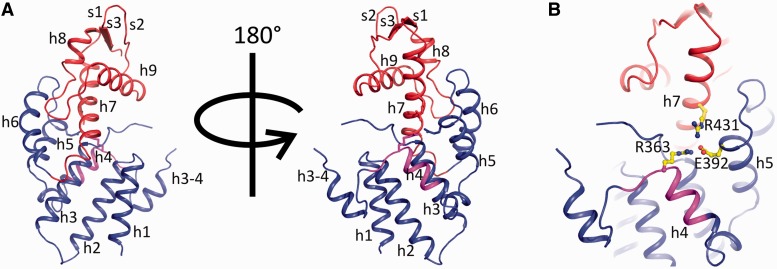
The overall structure of the PCI domain of a/TIF32. (**A**) Cartoon representation of a/TIF32^276–494^ crystal structure. The helical bundle and the winged helix motif are colored blue and red, respectively. Box37 (residues 361–370) within helix 4 is shown in magenta. α-helices and β-strands are denoted as h and s, respectively. (**B**) Interactions between HB and WH sub-domains. HB and WH are held together via hydrogen bonding between helices 4, 5 and 7 (h4, h5 and h7). Residues involved in these interactions are R363 (h4), E392 (h5) and R431 (h7) and are presented as sticks.

The crystal structure of a/TIF32^276–^^494^ consists of two intimately connected subdomains; an N-terminal right-handed helical bundle capped by a C-terminal winged helix (WH) domain (Figure 3A). The helical bundle (HB) is composed of three pairs of antiparallel helices with structural resemblance to tetratricopeptide repeats (TPR). Helices 3 and 4 are connected via a long loop and a short helix. Residues 342–344 of this loop are poorly visible in the electron density and therefore likely to be flexible. Interestingly, residues 337–362, which form the loop and the helix connecting helices 3 and 4, are not present in the structure of any of the known PCI domains. The winged helix sub-domain shows the canonical α-β-α-α-β-β topology with an N-terminal elongated helix, which is kinked at the beginning. The structure of the WH subdomain is well conserved among known PCI structures. The orientation of the WH relative to the HB is held by several hydrogen bonds and van der Waals interactions between helix 7 (the first helix of WH) and different helices from HB. Most notably, R431 of helix 7, E392 of helix 5 and R363 of helix 4 are tightly linked by hydrogen bonds locking these three helices in place (Figure 3B). R363 and the adjacent K364 together with R431 contribute to a positively charged surface on the concave face of the protein, proposing a function for this region in mRNA recruitment (see below). Y420 and L424 of helix 7 make van der Waals interactions with the side chain of L330 of helix 3. Y420 makes a hydrogen bond with H299 of helix 2, which also interacts with S331 of helix 3. Interestingly, all these residues are highly conserved among eukaryotes, suggesting that maintaining the relative orientation of WH and HB might have a functional relevance (Supplementary Figure S2).

**Figure 4.**
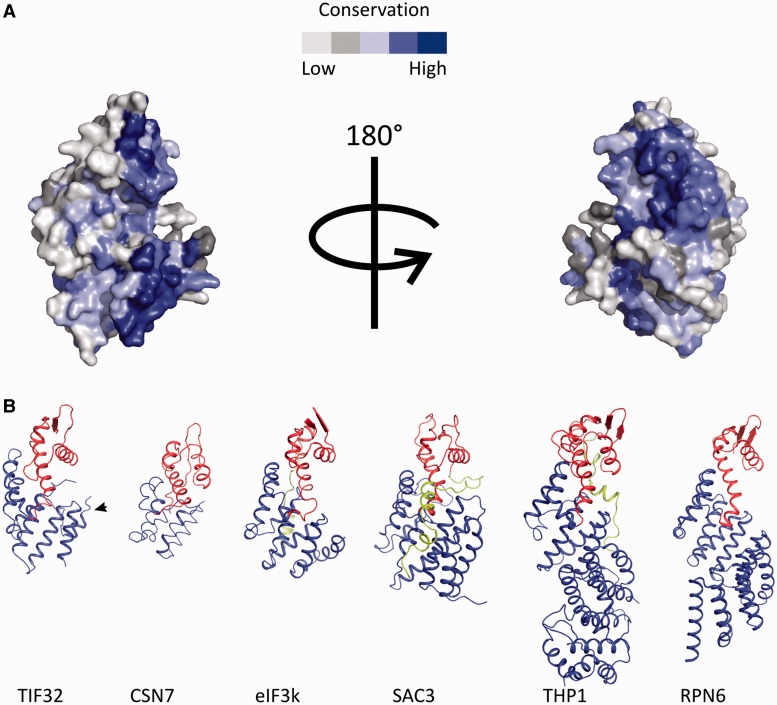
Conserved residues of a/TIF32 and comparison of the 3D structures of different PCI domains. (**A**) Conserved residues of a/TIF32. Surface representation of the conservation of residues among a/TIF32 from different organisms was performed using ConSurf server. (**B**) Comparison of the 3D structures of different PCI domains. In all structures, the HB and WH sub-domains are depicted in blue and red, respectively. C-terminal extensions of the WH, if present, are colored green. In the a/TIF32 PCI domain, the extra helix 3–4 is marked by an arrow.

In fact, a/TIF32 is one of the most conserved core eIF3 subunits among eukaryotes. Interestingly, the highest degree of conservation is observed in its N-terminal region, including the PCI domain (Supplementary Figure S2). The majority of the conserved residues were found to be in the hydrophobic core of a/TIF32^276–^^494^, pointing to their importance in the folding of this domain. We mapped the highly conserved residues on the surface of a/TIF32^276-494^ using ConSurf server (53). Although no region with particularly high degree of conservation was found throughout the surface, scattered regions of high conservation were found to be surrounded by less conserved residues mainly at the concave surface of the protein (Figure 4A).

### Comparison of the a/TIF32 PCI domain with other known PCI structures

Comparison of the structure of a/TIF32^276–^^494^ with PCI domains from RPN6 [proteasomal protein, PDB code 3TXN, (54)], CSN7 [COP9-signalosome subunit, PDB code 3CHM, (55)], eIF3k (eIF3 subunit, PDB code 1RZ4, (56)], SAC3 and THP1 [TREX-2 components, PDB code 3T5V, (35)] reveals high degree of structural similarity despite little sequence conservation (Figure 4B). The highest structural conservation is observed for the C-terminal WH subdomain, whereas the N-terminal HB is more divergent. In all these structures, HB sub-domains show an overall right-handed superhelical arrangement of α-helices with characteristics of both HEAT [**H**untingtin, elongation factor 3 (**E**F3), protein phosphatase 2A (PP2**A**) and the yeast kinase **T**OR1] and TPR helical repeats. While eIF3k has 11 helices, which form 3 HEAT repeats, and CNS7 consists of 6 helices forming a pair of HEAT repeats, one of which resembles an ARM motif, RPN6 and THP1 structures have longer N-terminal extensions of TPR-like repeats. RPN6 contains five pairs of TPR-like repeats forming a twisted solenoid arrangement, whereas Thp1 forms a straighter arrangement that is capped by a helical bundle at the N-terminus formed by four α-helices [for a review see (57)]. Our a/TIF32 structure consists of six helices with a superhelical arrangement. An extra helix connecting helices 3 and 4 is a novel feature of a/TIF32 compared with the other PCI domains. This region is relatively conserved among eIF3a from different eukaryotes.

### The PCI domain of a/TIF32 directly mediates its binding to c/NIP1

**Figure 5.**
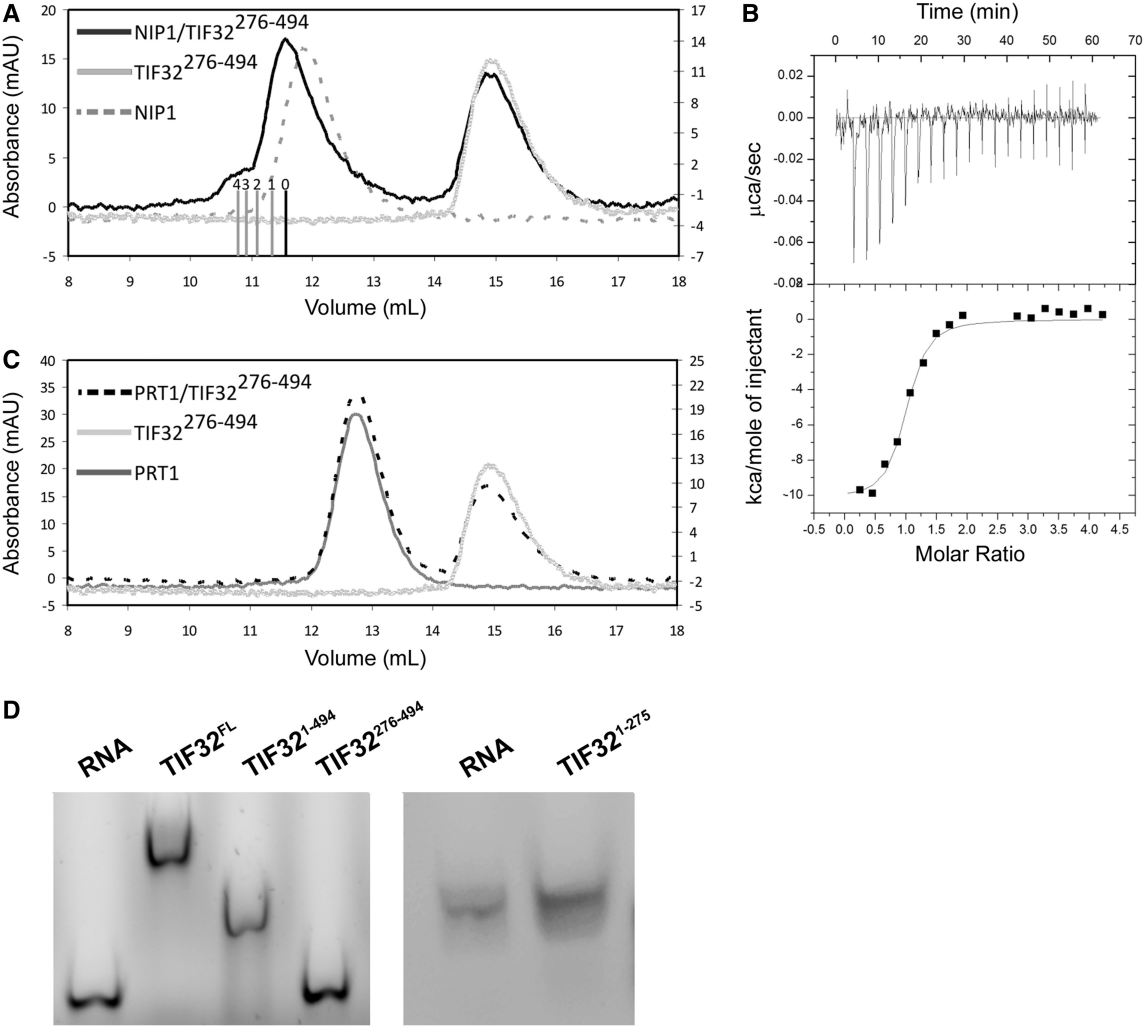
Protein and RNA-binding properties of a/TIF32^276-494^. (**A**) Interaction of a/TIF32^276–494^ with other major components of eIF3. SEC chromatogram of a/TIF32^276–494^ interaction with c/NIP1. The shift to the smaller volume in the case of the mixture of two proteins indicates a complex formation. Bars indicate different ratios of a/TIF32^276–494^ to c/NIP1, with 0 being only c/NIP1 and 4 being four molecules of a/TIF32^276–494^ bound to c/NIP1. The small peak at 10.8 ml after mixing a/TIF32^276–494^ and c/NIP1 is most probably an artifact of SEC due to incubation of the complex at room temperature for 30 min. (**B**) Isothermal calorimetric titration of c/NIP1 with a/TIF32^276–494^. The upper panel shows raw data of heat effect (in mcal s^−1^). The lower panel shows the fitted binding isotherms. The data points were obtained by integration of heat signals plotted against the molar ratio of a/TIF32^276–494^ to c/NIP1 in the reaction cell. The solid line represents a calculated curve using the best fit parameters obtained by a non-linear least squares fit. (**C**) The mixture of a/TIF32^276–494^ and b/PRT1 behaves the same as the sum of both proteins individually. (**D**) RNA binding activity of different regions of a/TIF32. Both full-length and the entire N-terminal domain of a/TIF32 bind to RNA. In contrast, a/TIF32^276–494^ or a/TIF32^1–276^ does not disturb the electrophoretic mobility of the mRNA.

PCI domains are known to be protein interaction sites (33). a/TIF32, the largest subunit of yeast eIF3, has been implicated in building the core of eIF3 by interacting with c/NIP1 and b/PRT1, two other large eIF3 subunits (18,58). We had previously purified a stable complex between a/TIF32^1–^^494^ and c/NIP1^244–^^812^ (45). To find out whether the PCI domain of a/TIF32 (276–494) *per se* mediates this binding, we performed analytical SEC with purified c/NIP1 and a/TIF32^276–^^494^. While each of the proteins elutes as a single peak from the column, the mixture of two proteins results in two peaks, with one having the same retention volume as a/TIF32^276–^^494^ alone (as it was used in excess over c/NIP1 to promote complex formation) and the other one shifting toward the smaller volumes compared with c/NIP1 indicating the formation of a complex (Figure 5A). To dissect the thermodynamics of this binding, we performed isothermal titration calorimetry (ITC) using a/TIF32^276–^^494^ and c/NIP1. The results indicated the formation of a complex with the stoichiometric ratio of nearly 1 (0.944), the dissociation constant (*K_d_*) of 158 nM and a relatively large enthalpy change of −10.23 kcal/mol (Figure 5B). As expected (59), no binding was detected between b/PRT1 and a/TIF32^276^^−^^494^ as judged by no change in the retention volumes of the mixture of the two proteins on analytical SEC compared with those of each protein alone (Figure 5C). Hence, the binding of a/TIF32 to b/PRT1 is most likely mediated via the C-terminal part of a/TIF32, as we recently also showed by *in vitro* reconstitution experiments (45).

### RNA-binding properties of the a/TIF32-PCI domain

Winged helix motives are well-known nucleic acid binding modules (60). It is therefore likely, but not necessarily self-evident, for a given PCI domain to modulate an interaction with nucleic acids. As mentioned previously, there are two PCI domains in yeast eIF3; one in the N-terminal region of a/TIF32 under study here and the other one in the C-terminal region of c/NIP1, which was recently implicated in RNA binding as the first PCI domain ever found to do so (34). To test whether the a/TIF32-PCI domain is also capable of binding to RNA, we performed electrophoretic mobility shift assay with different fragments of a/TIF32 and *DAD4* mRNA, which is one of the smallest naturally occurring mRNAs in yeast. A stretch of 30 adenosine nucleotides was added to the 3′-end of the mRNA during transcription to mimic the polyA tail of the mRNA. While full-length and the entire N-terminal domain (1–494) of a/TIF32 bound to the mRNA, a/TIF32^276–^^494^ failed to interact with the mRNA (Figure 5D). This may suggest an RNA-binding activity for the N-terminal region of a/TIF32 before residue 276. To check this, a/TIF32^1–^^276^ was purified as a GST-fusion protein and tested for binding to *DAD4* mRNA. However, this fragment also failed to bind to the mRNA. This can be explained in several ways; GST-a/TIF32^1–^^276^ is not properly folded, or the mRNA-binding site is masked by the GST or the RNA-binding site at the N-terminal region of a/TIF32 is distributed over a wide range of residues, both before and after residue 276. It has to be acknowledged here that the concentrations of RNA and protein used in this assay are relatively high and as such might produce non-specific RNA–protein interactions. However, this is of a minor concern as (i) only the entire N-terminal fragment of a/TIF32^1–^^494^ and not its N- and C-terminal halves interacted with RNA in our assay and (ii) a protein implicated in mRNA recruitment to ribosomes like a/TIF32 is expected to have general non-specific RNA-binding properties.

### The *tif32-Box37* mutation specifically diminishes mRNA recruitment to the 43S PICs *in vivo*

**Figure 6.**
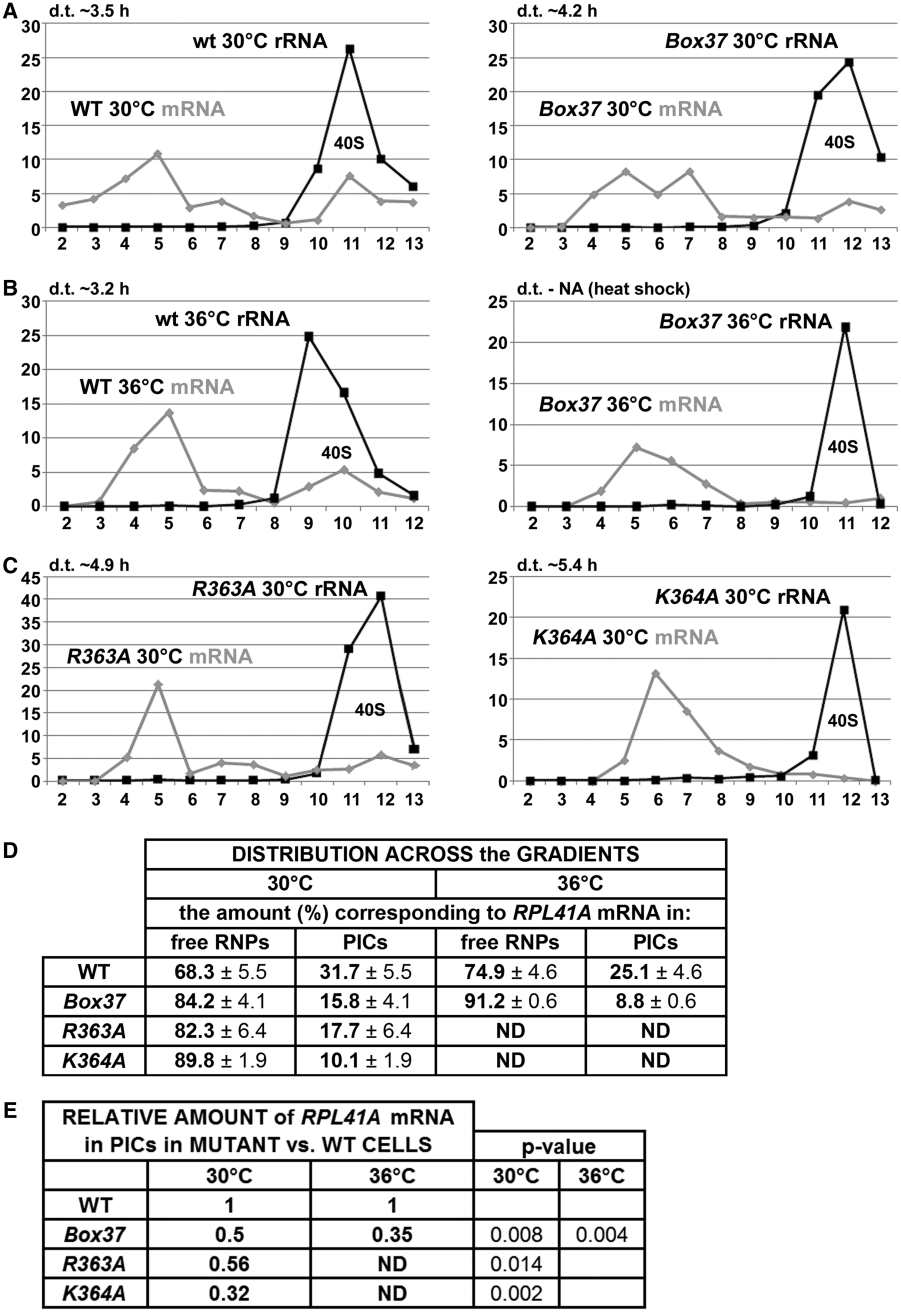
The *tif32-Box37*, *-R363A* and -*K364A* substitutions eliminate mRNA association with 43S PICs *in vivo*. (**A–C**) The isogenic *rpl11bΔ* strains carrying indicated *TIF32* mutations were cultured, cross-linked and separated as described in Figure 2. Total RNA was extracted from each of 12–13 fractions, and the amounts of 18S rRNA and *RPL41A* mRNA were measured by real-time quantitative polymerase chain reaction. The amounts of mRNA and 18S rRNA were calculated as 2^−Ct ^× 10^−7^ and 2^−Ct ^× 10^−4^, respectively. (**D** and **E**) The distribution of *RPL41A* mRNA across all fractions and its relative amounts in mutants versus wt in 18S rRNA containing fractions were calculated. Student’s *t* test (*P*-value) indicated that the value for each mutant differed significantly from that for the wt.

With several lines of experimental evidence and predictions from the 3D structure all pointing at the importance of the PCI domain for mRNA recruitment, we next decided to examine the effects of *Box37* on the 48S PIC assembly. To do that, we measured the amounts of three model mRNAs associated with native 48S PICs in whole-cell extracts of formaldehyde (HCHO) cross-linked cells that were resolved on sucrose gradients as described above according to (17). One of the model mRNAs—*RPL41A—*was successfully used in the latter and other studies, and the use of the other two—*DAD4* and *SME1—*was established here to provide broad specificity of our analysis. We specifically chose these mRNAs because they are rather short and thus minimize the ‘contamination’ effects of large mRNAs assembled into mRNPs that often run in heavy sucrose fractions like ribosomal species (17) and could lead to serious misinterpretations of the mRNA recruitment data. In addition, these experiments were conducted using isogenic strains lacking one of the genes encoding the 60S protein RPL11 (*RPL11B*), as the reduced level of 60S subunits resulting in a reduced rate of subunit joining increases the concentration of 48S PICs in *rpl11BΔ* cells (14). This is a necessary arrangement that increases the peak of mRNAs associated with the 48S PICs relative to the free mRNPs and significantly facilitates quantification of the 48S-bound species (17). Using real-time quantitative reverse transcription-polymerase chain reaction to assay the three reporter mRNAs and 18S rRNA in 12–13 collected fractions, we found that heat-shocked *Box37* significantly shifts distribution of all three mRNAs from 40S-containing fractions toward the top, indicating a substantial underutilized pool of free mRNPs (Figure 6A, B, and D, and Supplementary Figure S3). Relative amounts of all three mRNAs in PICs of mutant versus wt cells are shown in the corresponding Tables in Figure 6E and Supplementary Figure S3. This experiment was repeated several times with similar results. Taken together, we propose that the *tif32-Box37* 10-Ala substitution primarily impairs the assembly or stability of the 48S PICs. In other words, the α-helix 4 of the HB subdomain encompassing residues 361 through 370 markedly contributes to the stable mRNA recruitment to 43S PICs *in vivo*.

### Solvent-exposed K364 of the a/TIF32-PIC domain promotes mRNA recruitment

**Figure 7.**
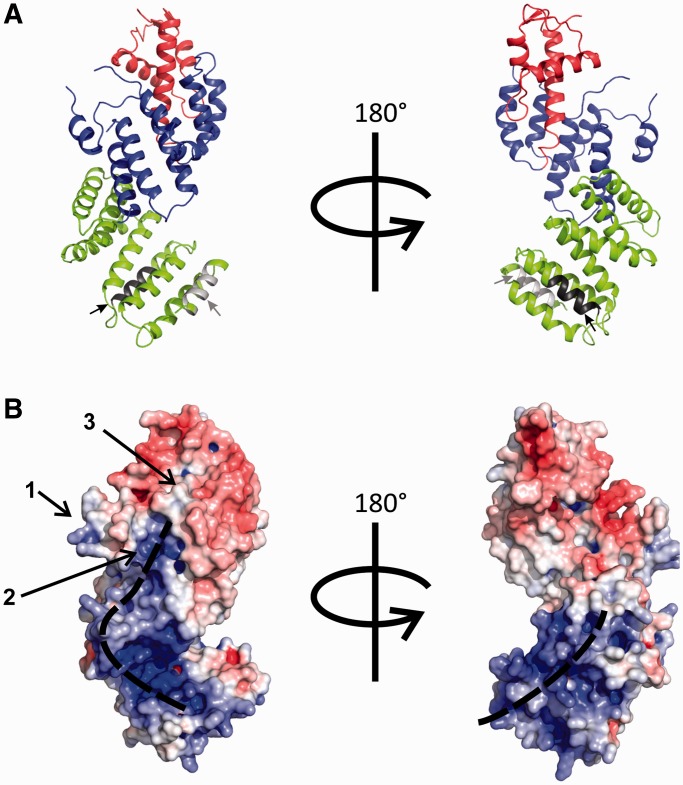
A proposed model of the entire a/TIF32-NTD. The crystal structure (residues 276–494) was extended by a homology-based prediction of residues 1–275. (**A**) The ribbon representation of the chimeric molecule. The WH and HB sub-domains of the crystal structure and the predicted helical extension are depicted in red, blue and green, respectively. Within the predicted region, Box6 (residues 51–60) and Box17 (residues 161–170) (see the text for details) are shown in light and dark gray, respectively, and are further marked by arrows of the same color. (**B**) Surface charge distribution of the chimeric molecule as calculated by APBS (61) plugin in PyMol (The PyMOL Molecular Graphics System, Version 1.0r1 Schrödinger, LLC.) shows the dominance of the basic side chains over the surface of the N-terminal helical extension (the predicted model). Two major patches of positive charges on opposite sides of the molecule are marked. Three major positive patches on the surface of the crystal structure are shown (left; 1, 2 and 3; the latter is created by the juxtaposition of residues R363, K364 and R431—see the text for details), which collectively form a basic area that aligns with the continuous basic patch on the front side of the N-terminal extension.

As mentioned previously, two N-terminal residues of the α-helix 4 of the HB subdomain (mutated in *Box37*) are R363 and K364. Whereas R363 is certainly required for stabilization of the HB fold (Figure 3B), K364 is fully exposed to the solvent with no structural role. However, both residues are part of the apparent continuous surface rich in basic residues with a fair probability to interact with RNA (region 3 in Figure 7B). Because the Ala-substitution of Box37 dramatically reduced the *RPL41A* mRNA amounts in the 40S-containing fractions *in vivo* (Figure 6A and B), it was tempting to speculate that these two residues actually mediate the mRNA transfer onto the 43 PICs. To address this question, we substituted both residues with alanine and examined the effects of these individual substitutions on *RPL41A* mRNA association with PICs *in vivo* as described above. As shown in Figure 6C–E and Supplementary Figure S4, both single substitutions decreased growth rates (by ∼2-fold) already at 30°C and, most importantly, markedly reduced the *RPL41A* mRNA amounts associated with the PICs. These observations thus strongly suggest that both residues play an important role in the ability of the PCI domain of a/TIF32 to stimulate formation of the 48S PICs. Interestingly, of the two, *K364A* had a stronger effect on mRNA recruitment (the relative amounts of *RPL41A* mRNA in PICs are reduced by ∼70%) as well as on the growth rates than *R363A* (showing ∼45% reduction in the relative amounts of *RPL41A*, the effect of which was comparable with the effect of *Box37*; see ‘Discussion’ section for further details). Taken together, we propose that one of the key roles of the a/TIF32-PCI domain is, besides building the eIF3 core, to contact mRNAs in a non-specific manner and promote their delivery to and/or stable binding on the small ribosomal subunit.

### Structural basis for the RNA binding of the a/TIF32-PCI domain

Secondary structure prediction suggests the dominance of helical repeats in the N-terminal region of a/TIF32, preceding the crystallized fragment. The sequence-based structure prediction using Phyre2 (62) suggests that the N-terminal HB probably extends further than six helices similar to the extended twisted character of THP1 and RPN6 (see above). Therefore, we built a chimeric molecule *in silico* from the predicted domain (residues 1–275) and the crystallized fragment (residues 276–494, Figure 7A). The relative orientation of the two domains was confirmed by performing protein–protein docking using HEX software (Supplementary Figure S5) (63). Analysis of the surface charge distribution of the predicted piece of the structure shows the dominance of positively charged patches, which form two continuous surfaces on the front and back of this N-terminal region of a/TIF32 implying a possible role in RNA binding (Figure 7B, left and right, respectively). Even though the crystallized fragment does not show an accumulation of charge density that would be comparable with a/TIF32^1–276^, the short α-helix connecting helices 3 and 4 of the PCI domain harbors several positively charged side chains, rendering it relatively basic (region 1, Figure 7B). Adjacent to this region is another patch of positive charge density on the concaved side of the structure made by K304, K305 and K308 of helix 2 (region 2, Figure 7B), followed by the last, only modestly basic region created by the juxtaposition of residues R363, K364 and R431 at the site where the HB joins the WH (region 3, Figure 7B). Together, these three regions constitute an overall basic area, which is, to some extent, aligned with the continuous positive charges on the surface of the modeled N-terminal fragment. Note that the first two of the latter residues from region 3 were experimentally implicated in promoting the mRNA loading to the 43S PICs *in vivo* (Figure 6). Moreover, 10-Ala substitutions of Box20 (the back RNA track of the NTD containing R194, R195 and K196), Box24 (the front RNA track containing R235), Box27 and Box29 (along the front RNA track below region 2 containing K268 and K288, respectively), Box31 (region 2 containing K304, K305 and K308) and Box35 (region 1 containing R350) produced lethal phenotypes (summarized in Figure 1B), indicating that they might be critical for mRNA recruitment. Although we cannot rule out a lot simpler explanation for the observed lethality—a defect in protein folding—the fact that no other Box mutants (with the only exception of Box38), including those in the PCI domain, were deleterious may speak in favor of a defect in RNA binding. Taken altogether, it is tempting to speculate that both the N-terminal and PCI domains of a/TIF32 promote mRNA recruitment in a co-operative manner, with the a/TIF32^1–^^276^ part probably making a bigger contribution to the overall binding. This is, in fact, consistent with our observations that neither the crystallized PCI fragment alone nor the N-terminal domain of a/TIF32 displayed any RNA-binding activity in solution, whereas the entire N-terminal half of a/TIF32 encompassing both of these domains did bind to the transcribed mRNA *in vitro* (Figure 5D).

## DISCUSSION

We reported previously that depletion of the entire eIF3 complex from cells impaired model *RPL41A* mRNA recruitment *in vivo* (14) and, furthermore, yeast eIF3 was later demonstrated to play a direct role in 43S PIC binding to capped, native mRNA *in vitro*, being even more critical than eIF4F and eIF4B (15). In this study, we showed that Ala substitutions in the α-helix 4 of the HB subdomain of the N-terminal PIC domain of a/TIF32, the crystal structure of which was solved at 2.65-Å resolution, robustly reduce the recruitment of three model mRNAs to the 43S PICs *in vivo* as the only detectable defect. Thus, these findings strongly suggest that this region of the a/TIF32-PCI significantly contributes to the formation of 48S PICs. In addition, our observation that several 10-Ala substitutions specifically mapping onto the mutually interconnected basic patches that we predicted to constitute the a/TIF32-NTD surface (Figure 7B; one of the patches includes the HB α helix 4) result in lethality further supports the idea that the entire N-terminal half of a/TIF32 is significantly involved in mRNA loading onto the 43S PICs, although we cannot rule out the protein folding effects. Importantly, in our recent study (17), substitutions in the KERR motif in the HLD located at the C-terminal half of the a/TIF32 were also shown to impair 40S-binding of model mRNA to the 43S PICs. However, in contrast to the PCI substitutions characterized here, the HLD substitutions also produced phenotypes indicating reduced efficiency of scanning and AUG recognition. Hence, to our knowledge, this is the first report implicating a specific domain of an initiation factor solely in this key role *in vivo*.

**Figure 8.**
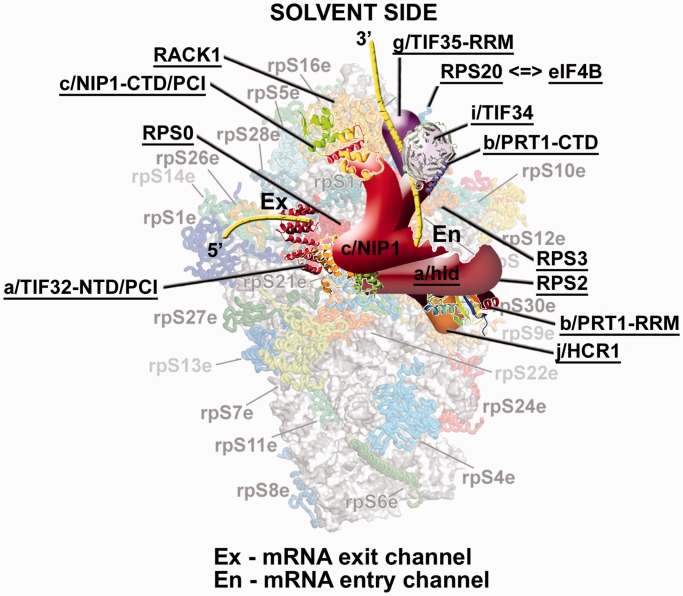
A model of eIF3 on the 40S ribosome spanning the mRNA exit and entry channels. The crystal structure of the 40S subunit is shown from the solvent side with ribosomal proteins shown as cartoons in individual colors; rRNA is shown as gray surface [adapted from (47)]. A hypothetical location of *Saccharomyces cerevisiae* eIF3 on the back side of the 40S subunit is based on the published interactions between RACK1 and the c/NIP1-CTD/PCI (34); RPS0 and the a/TIF32-NTD/PCI (2,46,64); RPS2 and j/HCR1 (24); RPS2 and 3 and the a/TIF32-CTD (17); helices 16-18 of 18S rRNA and the a/TIF32-CTD (64); and RPS3 and 20 and g/TIF35 (19). The extreme N- and C-terminal domains of c/NIP1 and a/TIF32, respectively, are predicted to interact with the interface side of the 40S subunit (64), as hinted. The recently published interaction between RPS20 and eIF4B is indicated by a double-headed arrow (69). Positions of all eIF3 subunits as well as RACK1, RPS0, 2, 3 and 20 are highlighted in bold. Schematic representations of i/TIF34 bound to the b/PRT1-CTD, the b/PRT1-RRM, the a/TIF32-NTD/PCI and the c/NIP1-CTD/PCI were replaced with the X-ray structures (25,30) or the 3D structural model (34), respectively. The yellow lines represent mRNA.

Actually, the additional post-assembly phenotypes associated with the HLD versus PCI substitutions can be most likely attributed to the fact that the CTD of a/TIF32 interacts *in vitro* with an 18S rRNA segment containing h16–h18 (64) and with RPS2 and RPS3 (17), all occurring nearby the mRNA entry pore on the solvent side of the 40S subunit (47) (Figure 8). It is believed that RPS3 plays a key role in opening and closing of the mRNA entry channel latch, stabilizing the closed position by interacting with h34 but interacting with h16 on the solvent side of the 40S subunit to promote the open-latch conformation (65). These latch movements are believed to play a critical role for the initial mRNA loading onto the 43S PICs and/or during the subsequent start codon selection process (65,66). Based on that, we naturally proposed that the a/TIF32-CTD can, by interacting with RPS3, h16 or h18, modulate the mRNA entry channel latch as a way of influencing the transition from open to closed PIC conformations during mRNA loading and scanning for AUG recognition (17). In support, hydroxyl radical cleavage mapping of mammalian eIF3 in the 48S PICs also suggested that proportions of its eIF3a and eIF3d subunits interact with h16 (67). Ribosomal location of the a/TIF32-PCI domain can be, on the other hand, predicted from the well-established interaction between RPS0A and the a/TIF32 segment spanning residues 201–400 (2,46,64). Incidentally, a significant contributor to this binding is the C-terminal tail of RPS0A, which is highly acidic (46), indicating an ionic character of its interaction with the basic a/TIF32-NTD. Consistently, a subunit of rabbit eIF3 was only recently predicted to contact RPS1 and 26 that are both situated next to RPS0 (29). Importantly, RPS0A occurs near the mRNA exit not the entry pore (Figure 8); i.e. relatively far from the ‘latch feature’ that thus should not be affected by the PCI mutations. In agreement, no phenotypes indicating dramatic post-assembly defects were observed using the *GCN4* translational mechanism as a genetic tool.

In detail, we found two basic residues (R363 and K364) located at the beginning of helix 4, which together with R431 of helix 7 constitute a positively charged patch on the surface of a/TIF32-PCI domain (Figure 7B, region 3). To test whether the mRNA recruitment defect observed for the Ala substitution of the entire helix 4 (in Box37) originates from the perturbation of this basic surface, each of these Box37 residues was mutated to Ala. Both *R363A* and *K364A* mutations did impair the recruitment of *RPL41A* mRNA to the 43S PICs *in vivo*, with the latter displaying a more pronounced defect than *Box37* and *R363A* (Figure 6). Attempts to purify the N-terminal half of a/TIF32 harboring *Box37*, *R363A* or *R363A K364A* mutations to examine the effects of helix 4 mutations on the *in vitro* RNA-binding property of the a/TIF32-NTD yielded poorly expressed and/or largely insoluble proteins in the bacterial expression strains that we used. Poor expression and insolubility could be attributed to problems in the folding of the a/TIF32-PCI domain, as R363 plays an important role in bringing helices 4, 5 and 7 together and maintaining the proper relative orientation of the WH and HB subdomains (Figure 3B). However, the folding problem in the context of the entire a/TIF32 would be most likely only minor provided that *Box37* dramatically affects neither the integrity of eIF3 nor the assembly of the MFC *in vivo*. Nevertheless, it is still possible that some minor misfolding stands behind the observed differences in the degree of impairment of mRNA recruitment in *Box37* and *R363A* versus *K364A* mutants. It could perhaps indirectly affect some step preceding mRNA binding that would thus become rate-limiting and specifically reduce the net effect on the mRNA recruitment impairment in *Box37* and *R363A* mutants. In contrast to R363, K364 is fully exposed to the solvent and therefore less critical for the folding of the a/TIF32-PCI domain, if at all. Consistently, its Ala substitution produced a soluble protein that, however, did not show any significant effect in our *in vitro* RNA-binding assay (data not shown). The ostensible paradox that *K364A* resulted in severe impairment of mRNA recruitment *in vivo* but did not affect the a/TIF32-NTD RNA binding *in vitro* could be explained by proposing that either our RNA-binding assay is not sensitive enough to detect the impact of *K364A* on the a/TIF32 ability to directly interact with mRNAs, or that the undisputable contribution of K364 to mRNA recruitment is indirect. In the latter case, it could be exerted in co-operation with other mRNA binding and recruitment-promoting eIFs, such as those associated with eIF4G, which most probably also bind to the solvent-exposed side of the 48S PICs as eIF3 does, at least based on their predicted locations [reviewed in (1)]. For example, this region of a/TIF32 could contact 18S rRNA (rather than mRNA) in such a way that it is not required to anchor eIF3 to the ribosome but to make 40S-bound eIF3 to adopt an ‘RNA-recruitment-competent’ conformation to enable stable binding of incoming mRNA-bound eIF4F complex to start scanning.

Along these lines, the ribosomal position of yeast eIF4B—a factor previously implicated in promoting ribosomal scanning by stimulating the helicase activity of eIF4A and by holding on to the single-stranded regions created by eIF4A (68)—has been only recently mapped. It was shown to interact *in vitro* with RPS20 (69) that is situated on the 40S head right above the mRNA entry pore (47) (Figure 8); its head-location around RPS20 was also corroborated by hydroxyl radical probing. The tight 40S-binding of eIF4B was then demonstrated to play a critical role in its novel unexpected role in promoting mRNA recruitment to 43S PICs. Interestingly, eIF4B interacts with g/TIF35 (70), earlier implicated in promoting processivity of scanning through stable secondary structures (19), and g/TIF35 in turn interacts with RPS20 and also RPS3 (19). Recently, we proposed that the scaffold b/PRT1 subunit serves to connect two eIF3 modules at each of its termini: (i) a/TIF32-HLD-CTD–j/HCR1 at the N-terminal RRM and (ii) i/TIF34–g/TIF35 at the extreme C-terminal α-helix, both of which surround the mRNA entry pore working together to fine-tune scanning and the AUG selection process (25) (Figure 8). Hence, it is tempting to speculate that although these two modules co-operate with eIF4B (via its contacts with g/TIF35 and RPS20) and possibly also with eIF4A in stimulating mRNA recruitment and promoting scanning for AUG, the a/TIF32-PCI co-operates with eIF4G—since time immemorial implicated in bridging the 40S–mRNA contact—to ensure mRNA loading onto the 43S PIC as their primary role. In fact, recent mutational analysis of yeast eIF4G showed that two of its functionally important RNA-binding elements, RNA2 and RNA3, do not critically contribute to formation of the eIF4G–PABP mRNPs suggesting that they could contribute to the function of eIF4G in recruitment of the 43S PIC to mRNAs (71), perhaps hand in hand with the a/TIF32-PCI and other factors. Even though the interaction between eIF3 and eIF4G has never been shown in yeast, in contrast to mammals (6,72), it does not mean that it cannot be established directly in the PIC or that eIF4G and a/TIF32-PCI could not work together without actually being bound to each other. In fact, earlier findings showing that several eIF3 subunits can directly interact with mRNA (2,19,34,67,73,74) may suggest that incoming mRNA is ‘grabbed by many hands’—some more, some less contributory—that could be physically and spatially independent of each other; however, collectively they would ensure proper and timely mRNA delivery to the mRNA-binding channel of the 40S ribosomes. Alternatively, as proposed before, the eIF3–eIF4G contact could be bridged by eIF5, which simultaneously interacts with eIF4G and with the extreme NTD of the c/NIP1 subunit of eIF3 (9,26). However, given the fact that eIF5 is most likely situated on the interface side of the 40S (75), whereas eIF4G is expected to occur on its solvent-exposed side [see (1) for the current view of factor’s occupancy on the 40S subunit], this possibility seems unlikely. Furthermore, the stimulatory function of eIF5 on mRNA recruitment to the PIC was not observed in a reconstituted yeast system (15) and a mutation in the eIF5-CTD that disrupts its interaction with eIF4G impaired 43S binding to mRNA only *in vitro* (9). Hence, the eIF5 contribution to this important initiation step, if any, awaits further examination. Without a doubt, detailed structural information about interactions of eIF3 subunits with the 40S ribosome, mRNA and other eIFs including eIF4B and eIF4G will be essential in deciphering molecular details of their critical roles in 43S attachment to mRNA.

Finally, we previously identified two N-terminal mutations in *tif32-Box6* and *17*, which produce the severe Gcn^−^ phenotype in an additive manner by disturbing the ability of 40S ribosomes to resume scanning after translating uORF1 to reinitiate on *GCN4* (36). Here we predict that they are both located close to two basic patches on the surface of the modeled N-terminal domain of a/TIF32 (Figure 7A). This supports our earlier proposal that both Boxes specifically contact reinitiation-promoting elements (RPEs) occurring in the 5′ UTR of uORF1 in the *GCN4* mRNA leader to stabilize the 40S subunit on uORF1 post-termination to resume scanning for efficient reinitiation downstream. Furthermore, it also indicates that in addition to this specific role, both segments may also non-specifically contact all cellular mRNAs and thus contribute to their recruitment to the 43S PICs.

## ACCESSION NUMBERS

The atomic coordinates have been deposited in the Protein Data Bank, www.rcsb.org (PDB ID code 4K51).

## SUPPLEMENTARY DATA

Supplementary Data are available at NAR Online.

## FUNDING

The Wellcome Trust [090812/Z/09/Z to L.S.V.]; Czech Science Foundation [305/10/0335 to L.S.V.]. Funding for open access charge: Wellcome Trust.

*Conflict of interest statement*. None declared.

## Supplementary Material

Supplementary Data
