# Supplementary material for: Structural integrity of the PCI domain of eIF3a/TIF32 is required for mRNA recruitment to the 43S pre-initiation complexes
Source: Nucleic Acids Res. 2014 Jan 13;42(6):4123–39. doi: 10.1093/nar/gkt1369 (PMC3973348; doi:10.1093/nar/gkt1369)
Supplement: Supplementary Data [file supp_42_6_4123__index.html]

Structural integrity of the PCI domain of eIF3a/TIF32 is required for mRNA recruitment to the 43S pre-initiation complexes — Structural integrity of the PCI domain of eIF3a/TIF32 is required for mRNA recruitment to the 43S pre-initiation complexes — Supplementary Data 

# Structural integrity of the PCI domain of eIF3a/TIF32 is required for mRNA recruitment to the 43S pre-initiation complexes

## Supplementary Data

files

**Files in this Data Supplement:**

- Supplementary Data - pdf file
