# Supplementary material for: Structural integrity of the PCI domain of eIF3a/TIF32 is required for mRNA recruitment to the 43S pre-initiation complexes
Source: Nucleic Acids Res. 2014 Jan 13;42(6):4123–39. doi: 10.1093/nar/gkt1369 (PMC3973348; doi:10.1093/nar/gkt1369)
Supplement: Supplementary Data [file supp_gkt1369_nar-01142-v-2013-File010.pdf]

SUPPLEMENTARY DATA

MATERIALS AND METHODS

Construction of yeast strains and plasmids

Strains YVV01, YVV02, YVV03 and YVV04 were generated from the strain WLCY06 (*MATa ura3-52 trp1-63 leu2-3,112 his4-301(AUU) tif32Δ::KanMX4 rpl11bΔ::LEU2* [YCp-a/TIF32-His-T])(1)as follows. WLCY06 was transformed with YCp-TIF32-His-U and grown in SD medium supplemented with excess of tryptophane to enable a spontaneous loss of YCp-a/TIF32-His-T. The resulting strain, auxotrophic for tryptophane, was named YVV01. YVV01 was then introduced with YCp-a/tif32-Box37-His-T, YCp-a/tif32-R363A-His-T or YCp-a/tif32-K364A-His-T, and the YCp-TIF32-His-U covering plasmid was evicted on 5-FOA producing YVV02, YVV03 or YVV04, respectively.

Derivatives of plasmid YCp-a/TIF32-His-screen with mutations *Box20* to *Box39* were generated by fusion PCR. For example, *Box20*was generated using YCp-a/TIF32-His-screen as template and primers LVTIF32-mut1 and LVTBOX20-1r in the first PCR reaction, and primers LVTBOX20-2 and LVTIF32-mut2-r in the second reaction. Primers LVTIF32-mut1 and LVTIF32-mut2-r were used in the third reaction with a 1:1 ratio of PCR products from the first and second reactions as template. The resulting PCR product was digested with *Bam*HI and *Hpa*I and inserted into YCp-a/TIF32-His-L digested with the same enzymes. Plasmids YCp-a/tif32-Box21-His to YCp-a/tif32-Box39-His were generated in a similar fashion using the appropriate primers (Table S3 shows primers only for Box37 that was used in this study).

Plasmids YCp-a/tif32-R363A-His and YCp-a/tif32-K364A-His were produced by fusion PCR using YCp-a/TIF32-His-screen as template. For both constructs primers LVTIF32-mut1 and SG82 were used in the first PCR reaction. Designated point mutation were introduced in the second PCR reaction using primers LVTIF32-mut2-r with combination of SG80 for YCp-a/tif32-R363A-His and SG79 for YCp-a/tif32-K364A-His. The third PCR reactions and cloning were done in the same way as for *Box* mutants.

YCp-a/tif32-Box37-His-T, YCp-a/tif32-R363A-His-T and YCp-a/tif32-K364A-His-T were created by ligation of *SphI-SacI* cut fragments carrying corresponding mutant alleles obtained from YCp-a/tif32-Box37-His-L, YCp-a/tif32-R363A-His-L and YCp-a/tif32-K364A-His-L,respectively, into YCplac22 which was digested using the same restriction enzymes.

Cloning, expression and purification of the a/TIF32$^{1-275}$and a/TIF32$^{276-494}$ domains

cDNAs encoding *TIF32*$^{276-494}$ and *TIF32*$^{1-276}$were cloned into the pGEX-6P-1 vector containing N-terminal GST tag (GE Healthcare). Both proteins were expressed in *E.coli* strain Rosetta2 (DE3) cells (Novagen) grown in auto-inducing medium (Studier 2005). Purification was done using a GSH-sepharose resin (GE Healthcare) equilibratedin 300 mM NaCl, 50 mM HEPES/NaOH (pH7.5), 5 % (w/v) glycerol and 2 mM beta-mercaptoethanol (bME). Proteins were eluted in the same buffer containing additional 30 mM reduced glutathione. The GST tag of a/TIF32$^{276-494}$ was removed using PreScission protease (GE Healthcare) while dialyzing against 200 mM NaCl, 30 mM HEPES/NaOH (pH 7.5), 5 % (w/v) glycerol and 2 mM bME overnight. GST, non-cleaved protein and the protease were removed by binding to GSH-sepharose

resin while a/TIF32$^{276-494}$ was collected as the flow-through. The protein was further purified on a superdex S-75 size-exclusion chromatography column (GE Healthcare) in 100 mM NaCl, 10 mM HEPES/NaOH and 2 mM DTT. To prepare selenomethionine containing protein, transformed Rosetta2 (DE3) cells were grown in a minimal media supplemented with selenomethionine (Studier 2005). Protein purification was conducted as for the native protein. c/NIP1, b/PRT1, a/TIF32 and a/TIF32$^{1-494}$ were purified as described previously (2).

Crystallization of the a/TIF32$^{276-494}$ domain and crystal structure determination

X-ray diffraction images were collected at 100 K at beamline 14.1 (BESSY, Berlin, Germany; (3)) equipped with a MAR Mosaic 225mm CCD detector (Norderstedt, Germany). The oscillation images were indexed, integrated, and merged using the XDS package (4,5) to the final resolution of 2.65 Å for the native and to 2.79, 2.81 and 2.97 Å for Se-Met derivative crystal (peak, inflection and remote datasets, respectively). The crystal structure of a/TIF32 was solved by means of SAD using the Se-Met dataset at the peak wavelength in SHARP/autoSHARP(6). Within autoSHARP the heavy atom search was performed by SHELXD (7) and resulted in localizing four heavy atom positions that were further refined using SHARP followed by density modification in Solomon (8) and automatic model building in Arp/wARP (9). The resulting protein model comprising 172 amino acids has been refined using torsion angle dynamics in CNS (10) and manually rebuild and verified in Coot (11) against Simulated Annealing (SA) omit maps. The model comprising 217 residues (from 6 to 223, including three methionines) and belonging to one a/TIF32 monomer has been refined using PHENIX (12) at resolution of 2.65 Å to R and R_{free} factors of 29.63% and 33.85%, respectively. Unusually high R-factors in concert with the solvent content of 72% and one additional (the fourth) Se peak found during the heavy atom search suggested the presence of additional protein molecule or its fragment in the asymmetric unit. The presence of the second full length a/TIF32 molecule in the asymmetric unit was unlikely due to the moderate resolution and resulting low solvent content of 42%. Performed MS analysis could not confirm any proteolytic digestion of protein samples obtained from dissolved crystals (data not shown). Difference electron density maps calculated with PHENIX (2mFo-DFc and mFo-DFc contoured at 1 and 3 sigma respectively) did not indicate any missing protein fragments as a few small separated blobs of density could not be interpreted as even a small part of a polypeptide chain. However the difference electron density maps (3mFo-2DFc, mFo-DFc) calculated with CNS revealed the presence of most probably some polypeptide fragments in the solvent channels, although the maps were highly diffused and not interpretable (no secondary structure elements could be recognized). In order to test if fragments of mostly α-helical a/TIF32 monomer could be the source of diffused electron density maps observed in solvent channels, we decided to carry out several molecular replacement (MR) searches with short a/TIF32 fragments as search models using PHASER (13) and keeping the already refined model as the fixed partial solution. About 650 short polypeptide fragments differing in length (25, 30, 35, 40, 55, 60 aa) have been generated with an offset of two and five amino acids covering the complete a/TIF32 monomer structure. The individual MR searches resulted in several well scoring solutions (TFZ scores between 9 to 11) which, when displayed simultaneously, formed an ensemble of overlapping fragments building a fragment of the second a/TIF32 molecule covering the amino acids range from 102 to 205. In order to localize the missing N-terminal fragment of

the second a/TIF32 molecule the search was repeated, this time using the refined a/TIF32 monomer and formerly found 103 amino acid long fragment of the second a/TIF32 molecule as the fixed partial model. Well scoring solutions could only be identified after increasing the value of RMSD from 0.35 Å (used for the first runs) to 0.5 Å. The necessity of increasing RMSD implicated higher level of positional disorder of the missing N-terminal part of the second a/TIF32 molecule in comparison to already localized 103 residues long fragment of it. Solutions with the highest TFZ scores (8 to 11) formed an ensemble comprising residues 4 to 65 of a/TIF32 monomer. Based on these results, two a/TIF32 fragments comprising residues 4 to 65 and 102 to 205, respectively have been subjected to additional molecular replacement search and were successful only when using RMSD of 0.9 Å and 0.6 Å for fragment one and two, respectively. The presence of the second a/TIF32 molecule was in addition confirmed by calculating the self-rotation function using GLRF (14) program giving one clear solution at 10 sigma level. The self-rotation axis corresponds to the rotation between two a/TIF32 monomers present in the asymmetric unit (the difference in kappa angle is 7 degrees). Refinement of the structure, comprising complete a/TIF32 monomer and two additional a/TIF32 fragments – residues 8 to 64 and 104 to 205, resulted in decrease of R and R_{free} factors to 24.85 % and 29.56 %, respectively. Due to high level of disorder of the second a/TIF32 molecule (average B-factor of 165 $Å^2$) reference model restrains generated from the complete a/TIF32 molecule, as implemented in PHENIX, have been used during the refinement. As a consequence no conformational differences can be observed between the two a/TIF32 molecules occupying the asymmetric unit of which only the complete a/TIF32 molecule has been refined independently.

RNA synthesis

A template for DAD4 RNA synthesis was prepared by PCR amplification from yeast genomic DNA using the following primers: GAAATTAATACGACTCACTATAAGCAGATAGGGAGGAAAAGAAGTGAGTTTA and ATGCGTATATAGAAAATTGGTGAATTAAA$(T)_{20}$. In the forward primer the sequence of the T7-promoter was included. A stretch of 20 T was added to the 5' end of the reverse primer to mimic the presence of a poly-A in the resulting RNA. The PCR product was precipitated with ethanol. To remove potential RNAse contaminations, Proteinase K was added and subsequently denatured by heating to 95°C. 1 – 1.5 mg DNA template was employed in an *in vitro* transcription approach containing T7 Polymerase and each 40 mM rNTPs in 1× HT buffer (30 mM HEPES pH 8.0, 25 mM $MgCl_2$, 10 mM DTT, 2 mM spermidine, 0.01% Triton X-100). After incubation at 37 °C for 3 h, the transcript was ethanol precipitated. The resulting pellet was dissolved in RNase-free water.

Other biochemical methods

β-galactosidase assays were conducted as described previously(15). Polysome profile analysis, 2% HCHO cross-linking, WCE preparation and fractionation of extracts for analysis of pre-initiation complexes were carried out as described by(16).

Analysis of the 48S PICs was done as described by(1)with the following exceptions. Total RNA was isolated from 0.5 ml of gradient fractions by hot-phenol extraction, and resuspended in 26 µl of diethyl pyrocarbonate (DEPC)-treated H_2O. Isolated total RNA was treated with 0.7 µl of DNaseI(NEB)in the total volume of 30 µl. 3 µl of RNA were subjected to reverse transcription with SuperScript III reverse transcriptase (Invitrogen) in the total volume of 20 µl. Aliquots of cDNA were diluted

3-fold or 12-fold for measuring mRNA or 18S rRNA levels, respectively (this way the cDNA was diluted 20 or 80-fold in total, respectively, in comparison to non-diluted RNA). qPCR amplifications were performed on 2 µl of diluted cDNA in 10-µl reaction mixtures prepared with the Brilliant II SYBR green qPCR Master Mix (Stratagene) and primers for *RPL41A* mRNA (0.3 µM), *DAD4* mRNA (0.3 µM), *SME1* mRNA (0.3 µM) or 18S rRNA (0.4 µM) using the Mx3000P system (Stratagene). For each round of qPCR, each fraction was measured in triplicates together with no-RT control. The experiment with each strain was performed at least three times for *RPL41A* mRNA and two times for *DAD4* and *SME1* mRNAs with similar results.

REFERENCES

1. Chiu, W.-L., Wagner, S., Herrmannová, A., Burela, L., Zhang, F., Saini, A.K., Valášek, L. and Hinnebusch, A.G. (2010) The C-Terminal Region of Eukaryotic Translation Initiation Factor 3a (eIF3a) Promotes mRNA Recruitment, Scanning, and, Together with eIF3j and the eIF3b RNA Recognition Motif, Selection of AUG Start Codons. *Mol Cell Biol*, **30**, 4415-4434.
2. Khoshnevis, S., Hauer, F., Milon, P., Stark, H. and Ficner, R. (2012) Novel insights into the architecture and protein interaction network of yeast eIF3. *RNA*, **18**, 2306-2319.
3. Mueller, U., Darowski, N., Fuchs, M.R., Forster, R., Hellmig, M., Paithankar, K.S., Puhringer, S., Steffien, M., Zocher, G. and Weiss, M.S. (2012) Facilities for macromolecular crystallography at the Helmholtz-Zentrum Berlin. *J Synchrotron Radiat*, **19**, 442-449.
4. Kabsch, W. (2010) Integration, scaling, space-group assignment and post-refinement. *Acta Crystallogr D Biol Crystallogr*, **66**, 133-144.
5. Kabsch, W. (2010) Xds. *Acta Crystallogr D Biol Crystallogr*, **66**, 125-132.
6. Vonrhein, C., Blanc, E., Roversi, P. and Bricogne, G. (2007) Automated structure solution with autoSHARP. *Methods Mol Biol*, **364**, 215-230.
7. Sheldrick, G.M. (2010) Experimental phasing with SHELXC/D/E: combining chain tracing with density modification. *Acta Crystallogr D Biol Crystallogr*, **66**, 479-485.
8. Abrahams, J.P. and Leslie, A.G. (1996) Methods used in the structure determination of bovine mitochondrial F1 ATPase. *Acta Crystallogr D Biol Crystallogr*, **52**, 30-42.
9. Langer, G., Cohen, S.X., Lamzin, V.S. and Perrakis, A. (2008) Automated macromolecular model building for X-ray crystallography using ARP/wARP version 7. *Nat Protoc*, **3**, 1171-1179.
10. Brunger, A.T. (2007) Version 1.2 of the Crystallography and NMR system. *Nat Protoc*, **2**, 2728-2733.
11. Emsley, P. and Cowtan, K. (2004) Coot: model-building tools for molecular graphics. *Acta Crystallographica Section D*, **60**, 2126-2132.
12. Adams, P.D., Afonine, P.V., Bunkoczi, G., Chen, V.B., Davis, I.W., Echols, N., Headd, J.J., Hung, L.W., Kapral, G.J., Grosse-Kunstleve, R.W. *et al.* (2010) PHENIX: a comprehensive Python-based system for macromolecular structure solution. *Acta Crystallogr D Biol Crystallogr*, **66**, 213-221.
13. McCoy, A.J., Grosse-Kunstleve, R.W., Adams, P.D., Winn, M.D., Storoni, L.C. and Read, R.J. (2007) Phaser crystallographic software. *J Appl Crystallogr*, **40**, 658-674.
14. Tong, L. and Rossmann, M.G. (1997) Rotation function calculations with GLRF program. *Methods Enzymol*, **276**, 594-611.
15. Grant, C.M. and Hinnebusch, A.G. (1994) Effect of sequence context at stop codons on efficiency of reinitiation in *GCN4* translational control. *Mol Cell Biol*, **14**, 606-618.
16. Nielsen, K.H. and Valášek, L. (2007) In vivo deletion analysis of the architecture of a multi-protein complex of translation initiation factors. *Methods Enzymol.*, **431**, 15-32.

17. Larkin, M.A., Blackshields, G., Brown, N.P., Chenna, R., McGettigan, P.A., McWilliam, H., Valentin, F., Wallace, I.M., Wilm, A., Lopez, R. *et al.* (2007) Clustal W and Clustal X version 2.0. *Bioinformatics*, **23**, 2947-2948.
18. Gouet, P., Courcelle, E., Stuart, D.I. and Metoz, F. (1999) ESPript: analysis of multiple sequence alignments in PostScript. *Bioinformatics*, **15**, 305-308.
19. Valášek, L., Trachsel, H., Hašek, J. and Ruis, H. (1998) Rpg1, the *Saccharomyces cerevisiae* homologue of the largest subunit of mammlian translation initiation factor 3, is required for translational activity. *J Biol Chem*, **273**, 21253-21260.
20. Munzarová, V., Pánek, J., Gunišová, S., Dányi, I., Szamecz, B. and Valášek, L.S. (2011) Translation Reinitiation Relies on the Interaction between eIF3a/TIF32 and Progressively Folded cis-Acting mRNA Elements Preceding Short uORFs. *PLoS Genet*, **7**, e1002137.
21. Nielsen, K.H., Szamecz, B., Valasek, L.J., A., Shin, B.S. and Hinnebusch, A.G. (2004) Functions of eIF3 downstream of 48S assembly impact AUG recognition and GCN4 translational control. *EMBO J.*, **23**, 1166-1177.
22. Gietz, R.D. and Sugino, A. (1988) New yeast-Escherichia coli shuttle vectors constructed with in vitro mutagenized yeast genes lacking six-base pair restriction sites. *Gene*, **74**, 527-534.
23. Valášek, L., Mathew, A., Shin, B.S., Nielsen, K.H., Szamecz, B. and Hinnebusch, A.G. (2003) The Yeast eIF3 Subunits TIF32/a and NIP1/c and eIF5 Make Critical Connections with the 40S Ribosome in vivo. *Genes Dev*, **17**, 786-799.

TABLES, FIGURES AND FIGURE LEGENDS

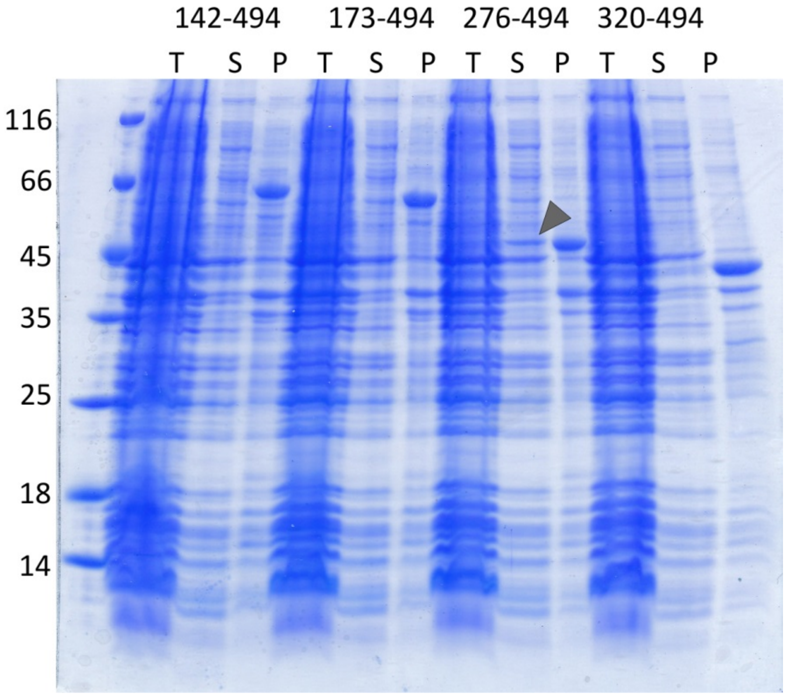

Supplementary Figure S1.Solubility test of different fragments of recombinant a/TIF32. Cell lysate was clarified by centrifugation, resulting in the soluble protein (S) and insoluble protein in the pellet (P). In each case, the total cell lysate prior to centrifugation is loaded on the gel (T). The best soluble fragment was a/TIF32$^{276-494}$ which is indicated by a black arrow.

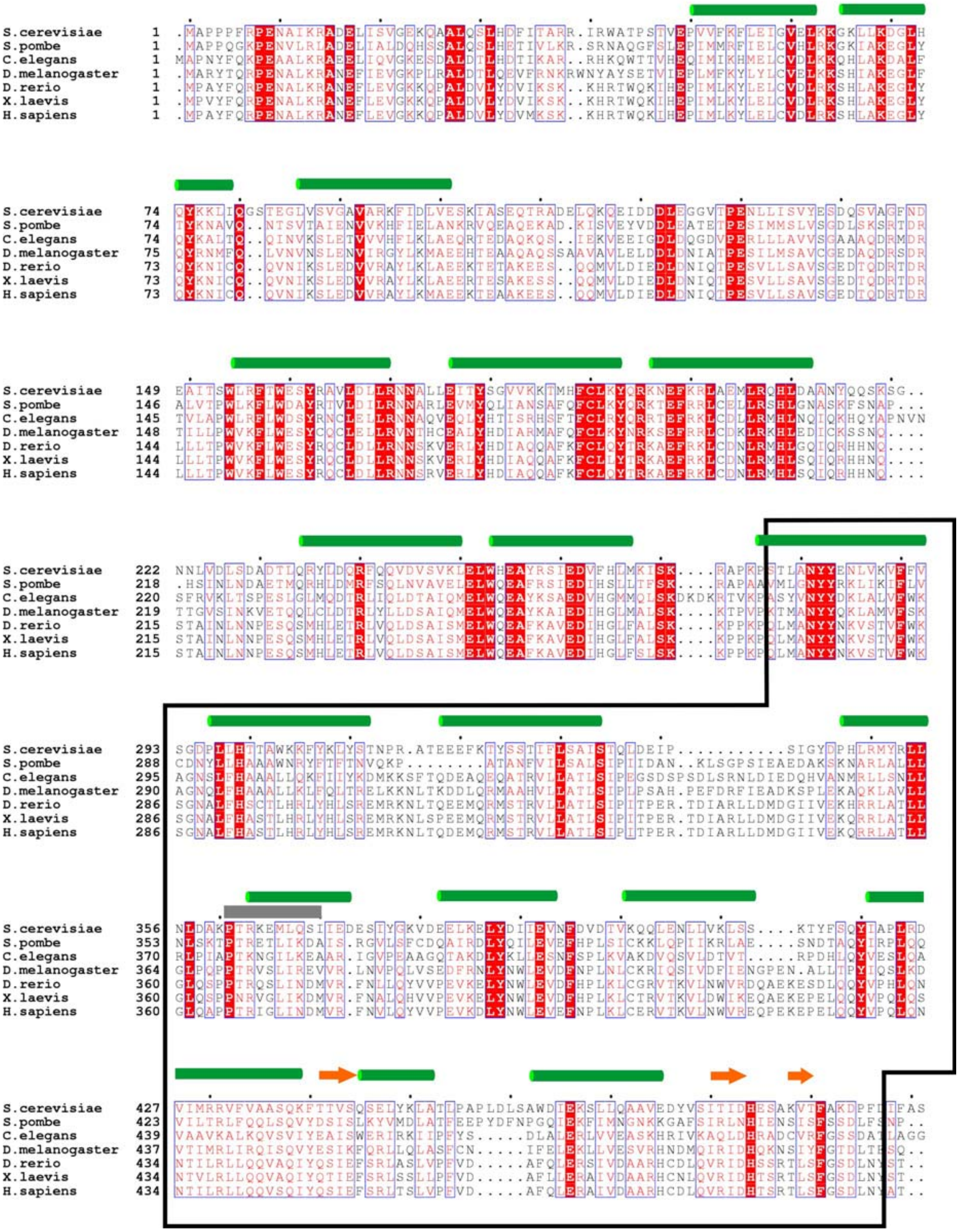

Supplementary Figure S2. Multiple sequence alignment of eIF3a/TIF32 from different organisms. Sequence alignment was done using ClustalW(17). Espript(18) was used for graphical presentation of the results. The crystallized fragment is marked by a box; Box37 is indicated by a grey bar. Green bars and orange arrows represent helices and strands, respectively.

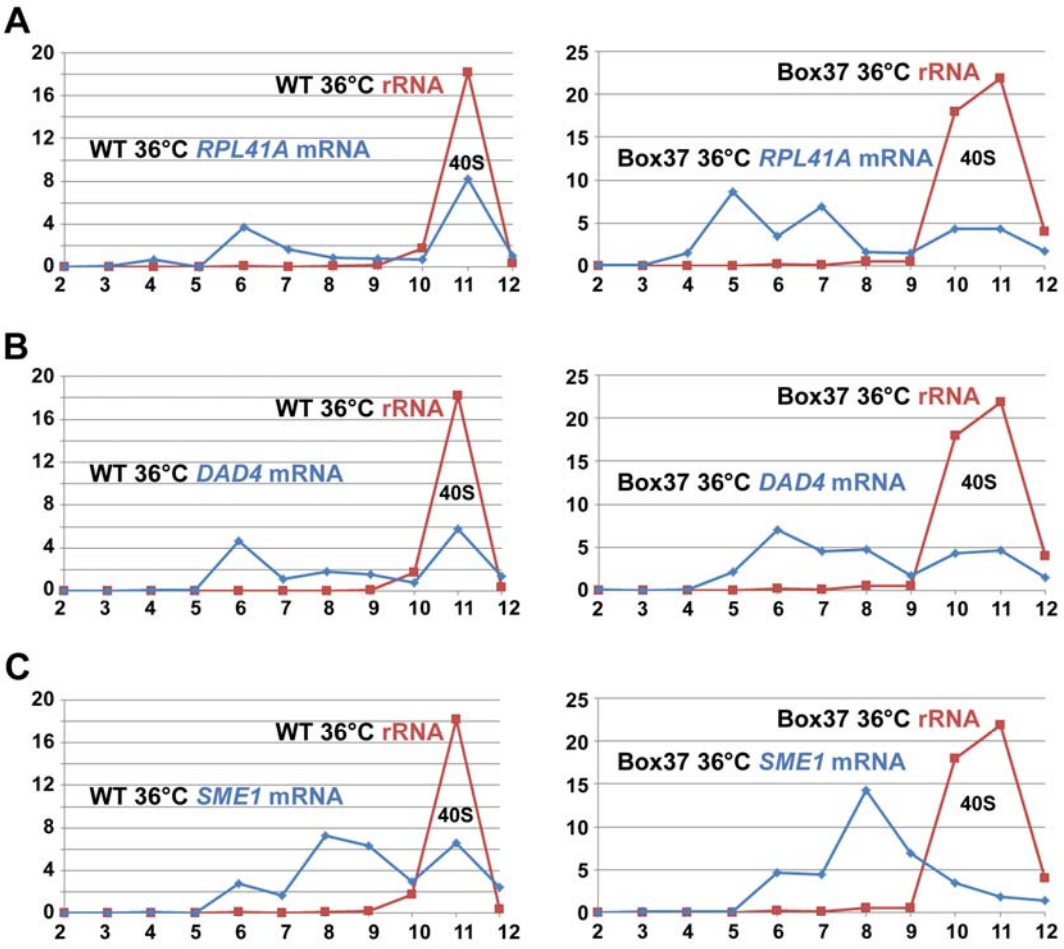

D

RELATIVE AMOUNTS of REPORTER mRNAs in PICs in MUTANT vs. WT CELLS at 36°C			
reporter mRNA	*RPL41A*	*DAD4*	*SME1*
WT	1	1	1
Box37	0.53 ± 0.03	0.64 ± 0.16	0.66 ± 0.1

Supplementary Figure S3. The *tif32-Box37* substitution eliminates association of three reporter mRNAs with 43S PICs *in vivo*. (**A – C**) The isogenic *rpl11bΔ* strains carrying either wt or mutant a/TIF32 were heat shocked at 36˚C for 4 hours and processed for mRNA binding analysis as described in Figure 6. The amounts of 18S rRNA and (**A**) *RPL41A*, (**B**) *DAD4*, and (**C**) *SME1* mRNAs were measured by real-time quantitative PCR (qPCR). (**D**) The relative amounts + SDs of all three mRNAs in the *tif32-Box37* mutant *versus* wt in 18S rRNA containing fractions were calculated.

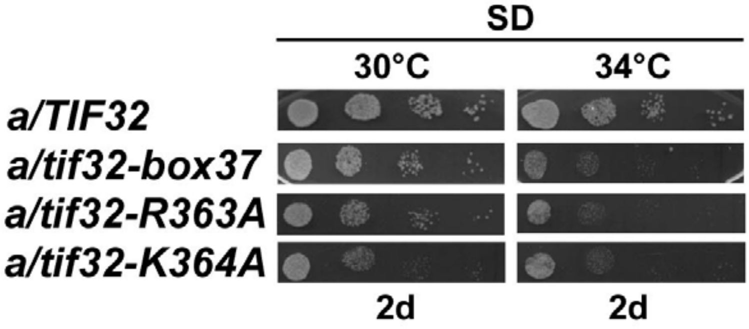

Supplementary Figure S4. The *tif32-R363A* and -*K364A* substitutions produce slow growth phenotypes. Isogenic strains WLCY06 (*a/TIF32*), YVV02 (*a/tif32-box37*), YVV03 (*a/tif32-R363A*) and YVV04 (*a/tif32-K364A*) were spotted in four serial dilutions on SD and incubated at 30°C or 34°C for 2 days.

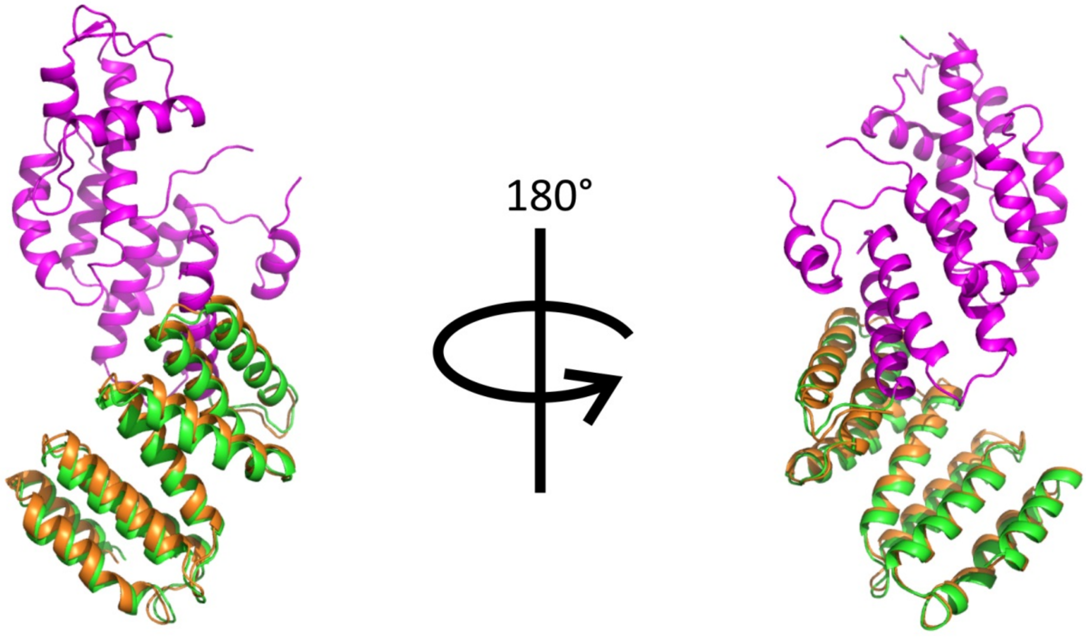

Supplementary Figure S5. Superimposition of the chimeric a/TIF32$^{1\text{-}494}$ built by merging the crystal structure (a/TIF32$^{276\text{-}494}$, magenta) and b/TIF32$^{1\text{-}275}$ as predicted by Phyre2 (orange) or HEX (green) programs.

Table S1. Yeast strains used in this study.

Strain	Genotype	Source or reference
H477	*MATa ura3::MET3-RPG1/TIF32 ura3 rpg1-del1::leu2::KanMX4 ade2-1 trp1-1 can1-100 leu2-112 his3-11 his3-15*	(19)
YBS52 [a]	*MATa leu2-3, -112 ura3-52 trp1Δ gcn2Δ tif32Δ URA3::GCN2 ura3* (YCp-a/TIF32-His-U)	(20)
H2880[a]	*MATa trp1Δ leu2-3,112 ura3-52*	(21)
H2881[a]	*MATa trp1Δ leu2-3,112 ura3-52 gcn2::hisG*	(21)
WLCY06 [b]	*MATa ura3-52 trp1-63 leu2-3,112 his4-301(AUU) tif32Δ::KanMX4 rpl11bΔ::LEU2* [YCp-a/TIF32-His-T]	(1)
YVV01[b]	*MATa ura3-52 trp1-63 leu2-3,112 his4-301(AUU) tif32Δ::KanMX4 rpl11bΔ::LEU2* [YCp-a/TIF32-His-U]	This study
YVV02 [b]	*MATa ura3-52 trp1-63 leu2-3,112 his4-301(AUU) tif32Δ::KanMX4 rpl11bΔ::LEU2* [YCp-a/tif32-Box37-His-T]	This study
YVV03 [b]	*MATa ura3-52 trp1-63 leu2-3,112 his4-301(AUU) tif32Δ::KanMX4 rpl11bΔ::LEU2 [*YCp-a/tif32-R363A-His-T]	This study
YVV04 [b]	*MATa ura3-52 trp1-63 leu2-3,112 his4-301(AUU) tif32Δ::KanMX4 rpl11bΔ::LEU2 [*YCp-a/tif32-K364A-His-T]	This study

[a,b]Isogenic strains.

Table S2. Plasmids used in this study.

Plasmid	Description	Source of reference
YCplac111	single-copy cloning vector, *LEU2*	(22)
YCp-a/TIF32-His-screen-L	single-copy *TIF32-His* with *Bam*HI and *Nde*I sites introduced just in front of the start codon of *TIF32* in *LEU2* plasmid, from YCplac111	(20)
YCp-a/tif32-Box37-His-L	single-copy *tif32-Box37-His* in *LEU2* plasmid, from YCplac111	This study
YCp-a/tif32-R363A-His-L	single-copy *tif32- R363A-His* in *LEU2* plasmid, from YCplac111	This study
YCp-a/tif32-K364A-His-L	single-copy *tif32- K364A-His* in *LEU2* plasmid, from YCplac111	This study
YCplac22	single-copy cloning vector, *TRP1*	(22)
YCp-a/TIF32-His-T	Single-copy *TIF32-His* in *TRP1* plasmid, from YCplac22	(1)
YCp-a/tif32-Box37-His-T	single-copy *tif32-Box37-His* in *TRP1* plasmid, from YCplac22	This study
YCp-a/tif32-R363A-His-T	single-copy *tif32- R363A* -His in *TRP1* plasmid, from YCplac22	This study
YCp-a/tif32-K364A-His-T	single-copy *tif32- K364A* -His in *TRP1* plasmid, from YCplac22	This study
YCp-a/TIF32-His-U	single-copy *TIF32-His* in *URA3* plasmid, from YCplac33	(23)

Table S3. Oligonucleotides used in this study.

Oligonucleotide	Sequence (5' to 3')
LVTBOX37-1r	CTTGGCGTCCAAGTTTAATAA
LVTBOX37-2	TTATTAAACTTGGACGCCAAGGCAGCTGCTGCAGCAGCAGCAGCAGCAGCAATTGAAGACGAATCCATTTAT
LVTIF32-mut1	CGCCCAGGAAGGATCCATATG
LVTIF32-mut2-r	TGTGTCGACATCAAAGTTAAC
SG79	CTTGGACGCCAAGCCAACTAGAGCGGAAATGTTGCAATCCATTATTGAAG
SG80	CTTGGACGCCAAGCCAACTGCAAAGGAAATGTTGCAATCCATTATTGAAG
SG82	GTTGGCTTGGCGTCCAAGT
RPL41A_f	CGAAATGAGAGCCAAGTGG
RPL41A_r	ATGCAATTTAGATCCATTATGAGG
DAD4_f2	CAAGTGCAGGCTAACATACTTTC
DAD4_r1	CGCTTCCAGGTTGAATTGGAC
SME1_f1	CGAGCAAATCGGCATAAGAATC
SME1_r1	CCTTCTCCACATCTTCTTTACC
18S rRNA_f	TCACCAGGTCCAGACACAATAAG
18S rRNA_r	TCTCGTTCGTTATCGCAATTAAGC
